# Comparison of measures of marker informativeness for ancestry and admixture mapping

**DOI:** 10.1186/1471-2164-12-622

**Published:** 2011-12-20

**Authors:** Lili Ding, Howard Wiener, Tilahun Abebe, Mekbib Altaye, Rodney CP Go, Carolyn Kercsmar, Greg Grabowski, Lisa J Martin, Gurjit K Khurana Hershey, Ranajit Chakorborty, Tesfaye M Baye

**Affiliations:** 1 Cincinnati Children's Hospital Medical Center, Department of Pediatrics, University of Cincinnati, Cincinnati, OH, USA; 2 Department of Epidemiology, University of Alabama at Birmingham, Birmingham, AL, USA; 3 Department of Biology, University of Northern Iowa, Cedar Falls, IA, USA; 4 Center for Computational Genomics, Institute of Applied Genetics, Department of Forensic and Investigative Genetics, University of North Texas Health Science Center, Fort Worth, TX, USA

## Abstract

**Background:**

Admixture mapping is a powerful gene mapping approach for an admixed population formed from ancestral populations with different allele frequencies. The power of this method relies on the ability of ancestry informative markers (AIMs) to infer ancestry along the chromosomes of admixed individuals. In this study, more than one million SNPs from HapMap databases and simulated data have been interrogated in admixed populations using various measures of ancestry informativeness: Fisher Information Content (FIC), Shannon Information Content (SIC), F statistics (F_ST_), Informativeness for Assignment Measure (I_n_), and the Absolute Allele Frequency Differences (delta, δ). The objectives are to compare these measures of informativeness to select SNP markers for ancestry inference, and to determine the accuracy of AIM panels selected by each measure in estimating the contributions of the ancestors to the admixed population.

**Results:**

F_ST _and I_n _had the highest Spearman correlation and the best agreement as measured by Kappa statistics based on deciles. Although the different measures of marker informativeness performed comparably well, analyses based on the top 1 to 10% ranked informative markers of simulated data showed that I_n _was better in estimating ancestry for an admixed population.

**Conclusions:**

Although millions of SNPs have been identified, only a small subset needs to be genotyped in order to accurately predict ancestry with a minimal error rate in a cost-effective manner. In this article, we compared various methods for selecting ancestry informative SNPs using simulations as well as SNP genotype data from samples of admixed populations and showed that the I_n _measure estimates ancestry proportion (in an admixed population) with lower bias and mean square error.

## Background

Admixture is a common form of gene flow between populations. It refers to the process in which two or more genetically and phenotypically diverse populations with different allele frequencies mate and form a new, mixed or 'hybrid' population [1,2]. A classic example of an admixed population in humans is the African-American population. As a result of the genetic admixture, the African-American population contains stretches of DNA as large as 20-30 cM that resemble mosaics of chromosomal segments, or ancestry blocks [3]. These segments are derived from intermixing between European and African ancestry and have not had sufficient time to break up through recombination [4,5]. As a result, in contrast to the million markers suggested to be necessary for genome-wide association studies (GWAS) [6], modeling studies showed that between 2000 and 5000 well-distributed ancestry informative markers (AIMs) distinguishing parental origins are sufficient for whole-genome scanning under the admixture mapping strategy [7-9]. An ideal AIM should have one allele that is fixed (i.e., allele frequency of 1.0) in one ancestral population, and not present in the other [10]. However, in the context of human genetics, most alleles are shared among populations [11-13]. Hence, it is important to identify and choose most ancestry informative markers across populations [14]; the power of admixture mapping relies heavily on the ability of informative markers to infer ancestry along the chromosomes of admixed individuals.

Several measures of marker informativeness for ancestry have been developed to select the most ancestry informative markers (reviewed in Rosenberg et al., 2003 [10]) from an ever-increasing wealth of genomic databases [15-18]. These measures include: Absolute Allele Frequency Differences (delta, δ), Shannon Information Content (SIC), Fisher Information Content (FIC), F statistics (F_ST_), and the Informativeness for Assignment Measure (I_n_). The cutoff value for δ is highly subjective and has steadily decreased over time from ≥ 0.5 [19] to 0.4 [20] to 0.3 [21,22]. Cutoffs that have been used for other measures are F_ST _≥ 0.4 [21], FIC ≥ 2.0 [10], SIC ≥ 0.3 [23], and I_n _≥ 0.3 [10]. Informative measures such as δ can be used for only two ancestral populations at a time [10,23]. On the other hand, F_ST_, FIC, SIC, and I_n _can be applied to select informative markers for admixed populations formed from two or more ancestral populations. For FIC and SIC indices, ancestral proportions in the admixed population need to be specified.

In spite of numerous studies with these measures of marker informativeness for ancestry, several questions are not systematically addressed, including how often are the same sets of SNPs selected by the different methods? To what degree do they overlap and share common sets of SNPs? How do AIM panels selected by these different methods perform in estimating ancestry population contributions under different proportion of ancestral population in an admixed population? With so many measures to choose from, it is very important to understand their common features as well as where they differ in terms of SNP selection. Answering these questions with a systematic study would help users in choosing appropriate measures in a cost-effective manner. In absence of a comprehensive comparative study on the performance of the different marker informativeness measures in marker selection, researchers selected markers using only the measure of their personal choice. For example, the three major U.S. admixture mapping research groups led by David Reich, Michael Seldin and Mark Shriver in their recent independent admixture mapping panels for Latino populations used SIC, F_ST _and δ [9,24,25], respectively. It is not clear which measure-based panel is the most informative for admixture mapping. In particular as more and more markers become available and as we study less differentiated populations, it is inevitable that prioritizing the most informative markers for ancestry inference or admixture mapping is critical. With the availability of common sets of SNPs from HapMap populations, we are given the opportunity to compare these methods directly. The objective of the present study is to compute and compare the commonly used measures of informativeness to select AIM panels for admixture mapping and structured association testing for admixed populations. To compare these methods, simulated as well as real data were used. In the simulated data, the ancestral populations and their contributions for each individual are known, allowing comparison of accuracy of the different measures of marker informativeness for ancestry using true and estimated individual ancestry values.

## Results

### SNP allele frequencies and comparisons of informative marker selection measures

**Figure 1:**
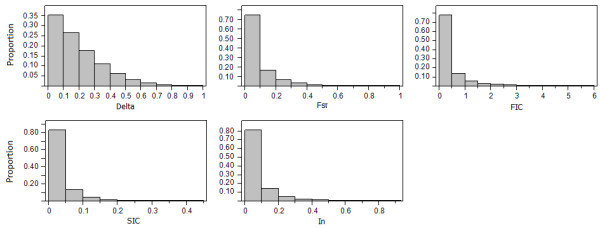
**Distribution of the five measures of marker informativeness for CEU and YRI population from HapMap phase III data**. The majority of the SNP markers display low to moderate estimates of genetic informativeness with few markers displaying high levels of population differentiation.

There are 1,362,723 and 1,450,896 autosomal SNPs in HapMap phase III release #3 dataset for CEU and YRI population, respectively. Table 1 shows, by chromosome and across the genome, the number of SNPs genotyped in each population and shared by both populations. After removing SNPs that did not meet our criteria (common in both YRI and CEU population and SNPs with missing frequency less than 10% of the samples), we found 1,264,741 SNPs shared by the CEU and the YRI datasets. Furthermore, to avoid the possibility of choosing two redundant SNPs that are in strong LD (linkage disequilibrium), for each measure, we calculated the informativeness on all shared SNPs, then filtered them for the most informative ones such that the physical distance between consecutive selected SNPs must be at least 100 kb. With this final filtering constraint and without using any cutoffs for any measures, all five measures gave AIM panels of size ~19.8 k. Figure 1 shows the distribution of the five measures of marker informativeness. A predominantly right-skewed distribution was produced for each selection method. Summary statistics of the five measures of marker informativeness are shown in Additional file 1, Table S1. The means of δ, F_ST_, FIC, SIC, and I_n _were 0.19, 0.07, 0.35, 0.03, and 0.06, respectively. The majority of the markers contained a small amount of ancestry information, suggesting a very high similarity in allele frequencies among common variants (frequency > 5%) in CEU and YRI population.

**Table 1:** Chromosome length and number of SNP markers across the genome in CEU and YRI population of the HapMap Phase III dataset

		Number of SNPs			SNPs genotyped in both population^a^				
Chr #	length	in each population		SNPs genotyped	and fulfilling the filtering criteria^b^				
	(Mb)	CEU	YRI	in both populations^a^	Delta	F_ST_	FIC	SIC	I_n_
1	246.6	111887	120349	103330	1663	1657	1653	1651	1654
2	242.7	113613	122377	106053	1742	1751	1742	1740	1749
3	199.3	94608	101070	88060	1464	1447	1448	1450	1450
4	191.2	85403	92052	79856	1390	1394	1387	1398	1392
5	180.6	87071	92350	81083	1310	1298	1314	1302	1304
6	170.7	91415	95108	84536	1241	1235	1239	1231	1236
7	158.7	75234	79231	69766	1139	1144	1152	1157	1148
8	146.2	74443	79368	69177	1053	1055	1053	1048	1054
9	140.2	63507	66437	58713	842	841	834	844	845
10	135.3	72846	76787	67388	979	988	980	980	988
11	134.3	69175	73942	64140	986	987	988	990	981
12	132.3	67486	70909	62018	988	981	973	972	977
13	96.2	51879	55392	48496	719	726	701	713	723
14	88.2	44570	47354	41434	646	646	650	648	650
15	82.0	40705	43837	37774	590	596	596	590	595
16	88.7	42738	46366	39616	568	559	554	555	557
17	78.6	36534	39075	33576	572	569	580	574	570
18	76.1	40153	43467	37669	562	568	564	561	572
19	63.6	25251	26798	23264	405	404	404	407	405
20	62.4	35252	37476	32870	447	447	446	448	443
21	37.1	19336	20556	18105	252	250	254	249	249
22	35.1	19617	20595	17817	249	253	253	252	249

Total		1362723	1450896	1264741	19807	19796	19765	19760	19791

^a ^Two inclusion criteria are in effective: 1) The SNP is shared by both YRI and CEU populations and 2) SNPs with missing frequency less than 10% of the samples.

^b ^Every SNP is at least 100 kb from its nearest neighbor.

For CHB and JPT population, the distribution of the five measures of marker informativeness is show in Additional file 2, Figure S1 and summary statistics are shown in Additional file 3, Table S2. Almost all the means of the five measures were about 10-fold less than those from the CEU and YRI population, indicating that CHB and JPT are much less differentiated than CEU and YRI and the ancestry estimation for an admixed population from CHB and JPT presents a much more difficult problem.

### Correlation, concordance, and overlapping analysis

#### Spearman correlation

**Figure 2:**
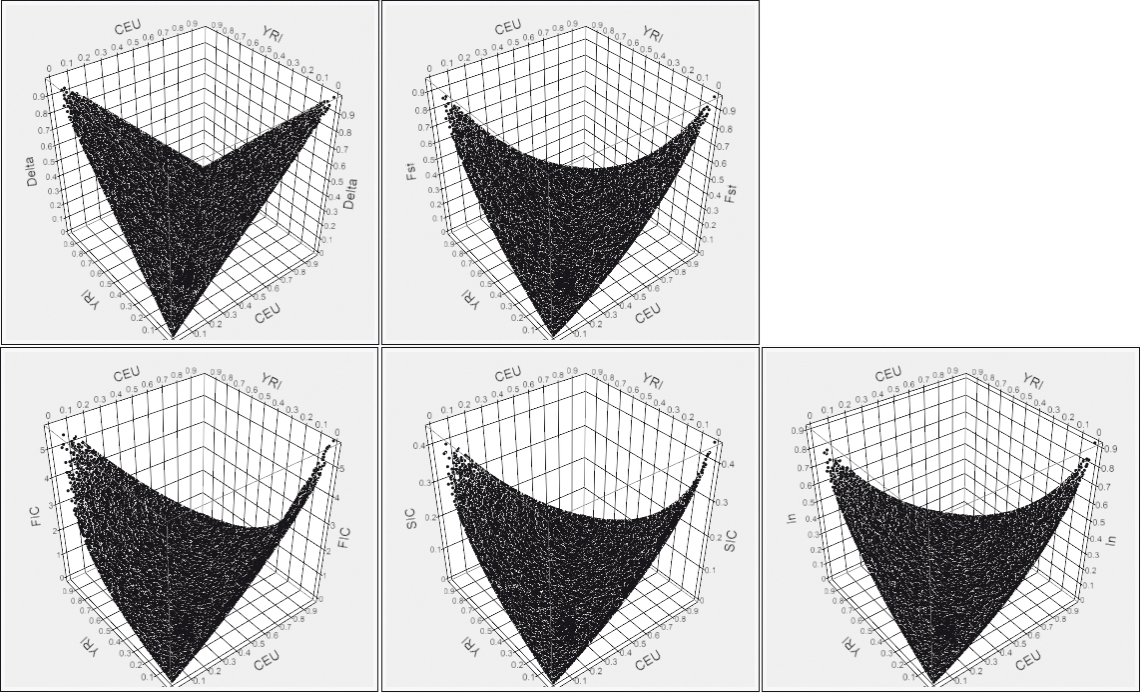
**3D scatter plot of CEU and YRI allele frequencies and the five measures of informativeness**. The two horizontal axes are frequencies of alleles shared by the two populations and the vertical axis is the calculated values of marker informativeness for ancestry by the five different measures. Similar symmetric patterns were observed between F_ST _and I_n_, and FIC and SIC exhibited somewhat asymmetric patterns.

**Figure 3:**
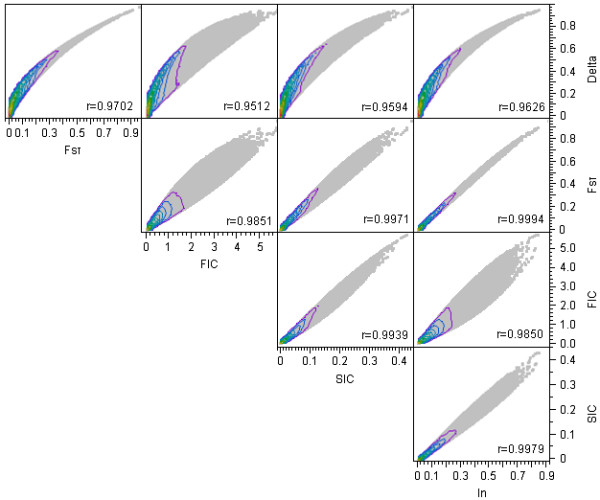
**Scatter plots of the five measures of marker informativeness with nonparametric quantile density**. In each panel, r is the Spearman correlation coefficient, which ranged from 0.9512 between δ and FIC to 0.9994 between F_ST _and I_n_. F_ST _and I_n _had an almost perfect monotonically increasing relationship.

To assess the level of similarity of the estimates of genetic information contained in each SNP marker across the different selection methods, Spearman correlation coefficient was calculated for the estimates of informativeness from different selection methods for CEU and YRI population. Figure 2 shows 3D scatterplots of CEU and YRI allele frequencies and the five measures of informativeness. Extremely similar symmetric patterns were observed between F_ST _and I_n_, whereas FIC and SIC exhibited somewhat asymmetric patterns. Pairwise scatterplots of the five measures of informativeness showed that the measures had high levels of correlation (Figure 3), with Spearman correlation coefficients ranging from 0.95 between δ and FIC to 0.99 between F_ST _and I_n_. F_ST _and I_n _had an almost perfect monotonically increasing relationship.

#### Concordance by deciles

**Figure 4:**
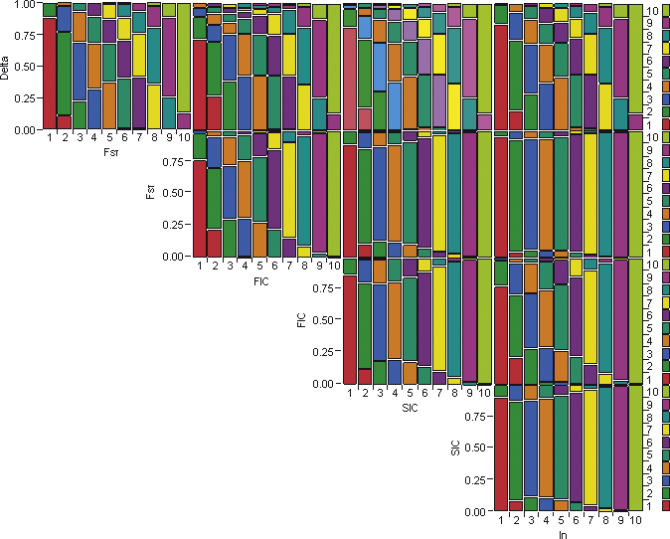
**Mosaic plot of the five measures of marker informativeness grouped by their deciles**. Each mosaic plot was first divided horizontally into ten bars with equal width representing the ten groups of a measure of informativeness. The first group (left most bar) contained the SNPs with highest information and the last group (right most bar) contained the SNPs with lowest information. Each bar was then split vertically into different colored segments whose heights were proportional to the probabilities associated with the second measure of informativeness, conditional on the first measure. Higher concordance was observed at the two ends of the informative scale.

Figure 4 shows mosaic plots of the five measures grouped by their deciles. The concordance at the two ends of the informative scales was higher than that in the middle of the scale. For example, most SNPs with values in the first group of informative scales for F_ST _also fell into the first group of δ, with a few falling into the 2^nd ^group of δ. However, even though some of the SNPs in the 2^nd ^group of FIC fell into the first group of δ, some in that same group fell into as high as the 6^th ^group of δ. The high concordance at the edges of the mosaic plots may be due to an edge effect. Again F_ST _and I_n _showed very high concordance, which indicates the ability of the two measures to identify AIM SNPs in a similar manner. This high similarity in picking informative markers can also be seen by the high correlation coefficients between these two measures (Figure 3). Delta had relatively poor concordance with the other four measures of informativeness. Kappa statistics (Additional file 4, Table S3) of the five measures of informativeness grouped by deciles further confirmed the above observations. F_ST _and I_n _had the best agreement (kappa = 0.93). F_ST _and SIC, and SIC and I_n _also showed good agreement, with the Kappa statistics of 0.85 and 0.86, respectively. Delta, with Kappa statistics between 0.42 and 0.47, had relatively poor agreement with the other four measures.

Additional file 5, Figure S2 shows the scatter plot of allele frequencies of CEU and YRI, with different colors indicating which decile group a SNP fell into. It is interesting to see how the ten groups partitioned the SNPs into symmetric patterns from the top-left and bottom-right corner representing the most informative SNPs to the center of the plot where the least informative SNPs resided. It is evident that δ showed a partition pattern different from other four measures due to the fact that δ depends only on the difference between allele frequencies of the two populations. F_ST _and I_n _exhibited very similar partition patterns, which is consistent with what we observed using the Spearman correlation coefficient, Mosaic plots, and Kappa statistics. It can also be recognized through the scatter plot that FIC favors the selection of markers that are closer to fixation in one of the populations.

#### Overlapping

**Figure 5:**
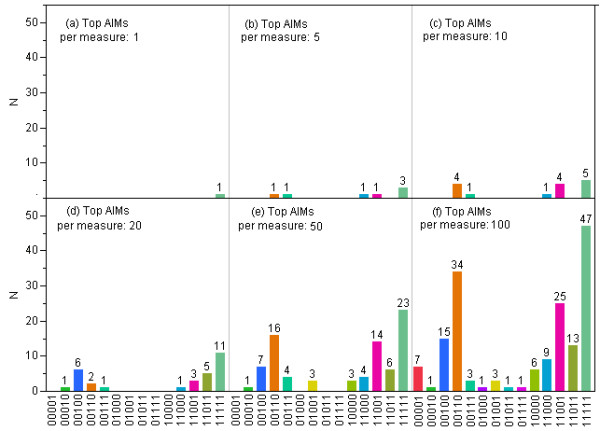
**Overlap of top *n *AIMs selected by different measures of informativeness**. For (a) n = 1, (b) n = 5, (c) n = 10, (d) n = 20, (e) n = 50, and (f) n = 100, a 5-digit binary vector was assigned to each SNP, where each digit represents a measure, and they are, from the first to the last, Delta, F_ST_, FIC, SIC, and I_n_, respectively. A 1 in a digit indicates that the SNP was selected by the corresponding measure as one of the top *n *AIMs.

Figure 5 shows overlap of top *n *(*n *= 1, 5, 10, 20, 50, and 100) AIMs selected by different measures of informativeness. For *n *= 1 (Figure 5a), the five measures selected the same SNP as the top AIM. For *n *= 10 (Figure 5c), there were totally 15 SNPs selected by one or more of the five measures. Five of them were selected by all the five measures, 4 were selected by FIC and SIC, and 4 were selected by δ, F_ST_, and I_n_. It can be seen across different *n *that, a relatively larger number of AIMs were selected by all five measures simultaneously, FIC and SIC were more likely to pick the same set of SNPs, and δ, F_ST_, and I_n _were more likely to pick the same set of SNPs. As the number of top AIMs increased, FIC was more likely to choose SNPs that were not chosen by any other measure.

### Discrimination analysis and estimation of ancestral contribution

#### Discrimination analysis

**Figure 6:**
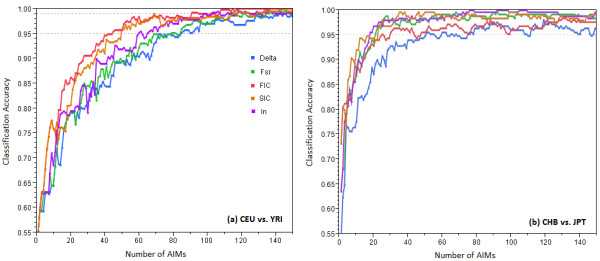
**Classification accuracy for ancestral population vs. number of top AIMs used by the five measures of informativeness**. (a) CEU vs. YRI and (b) CHB vs. JPT population.

Figure 6 shows classification accuracy vs. the number of top AIMs used by different measures of informativeness. For CEU vs. YRI population (Figure 6a), top AIMs chosen by FIC and SIC performed the best, while those selected by δ performed the worst. For CHB vs. JPT population (Figure 6b), δ performed the worst, while the other four measures performed comparably. Additional file 6, Figure S3 shows the number of top AIMs needed by each measure to achieve 90% or 95% classification accuracy. More AIMs were needed to 100% correctly differentiate individuals from CHB or JPT population than individuals from CEU or YRI population.

#### Estimation of ancestral contribution in admixed populations with top AIMs

Additional file 7, Figure S4 shows an inferred population structure (STRUCTURE [26] and *distruct *[27]) for CEU, YRI, and ASW population with the top 200 AIMs selected by FIC. AIM panels by other measures gave the same population structure. Furthermore, Additional file 8, Figure S5 shows the estimates of ancestry contributions for the three populations as the number of AIMs increases. The top informative SNPs (100 to 200) obtained using each measure yielded similar estimates of ancestry contribution. With 200 AIMs, the estimate of ancestry in ASW was 78% YRI across all measures. However, for individuals from CEU and YRI population, where the estimated ancestry contribution from CEU and YRI, respectively, is expected to be or close to 1, AIMs selected by I_n _performed slightly better than those selected by other measures of informativeness.

**Figure 7:**
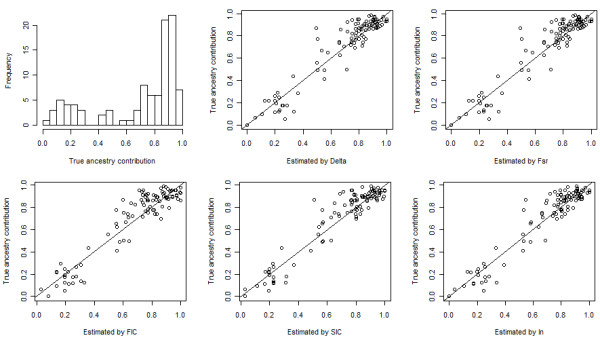
**Individual true ancestry contributions and estimated contributions using top 20 AIMs of the simulated admixed population from CEU and YRI**. Top-left panel: histogram of individual true ancestry contributions. Top-middle panel to bottom-right panel: scatter plot of individual true ancestry contributions vs. individual estimated contributions using top 20 AIMs selected by Delta, F_ST_, FIC, SIC, and I_n_, respectively.

For the simulated admixed population from CEU and YRI, a random sample of 100 individuals was extracted. The true average ancestry contribution was 70:30. Additional file 9, Figure S6 (a) shows absolute errors in the estimation of the ancestry contribution for the simulated population with 20, 50, and 100 top AIMs selected by different measures of informativeness. The lowest errors were achieved at either 20 or 50 AIMs by the methods. The bias in the estimation of the mean ancestry contribution was 0.01 by I_n _with 20 AIMs, 0.02 by SIC and FIC with 20 AIMs, and 0.01 by F_ST _and Delta with 50 AIMs. Using the top 20 AIMs, RMSE's were 0.095, 0.095, 0.100, 0.093, and 0.089 for δ, F_ST_, FIC, SIC, and I_n_, respectively. Figure 7 shows the histogram of the individual true ancestry contributions and the scatter plot of individual estimated contributions vs. true contributions with top 20 AIMs selected by different methods.

**Figure 8:**
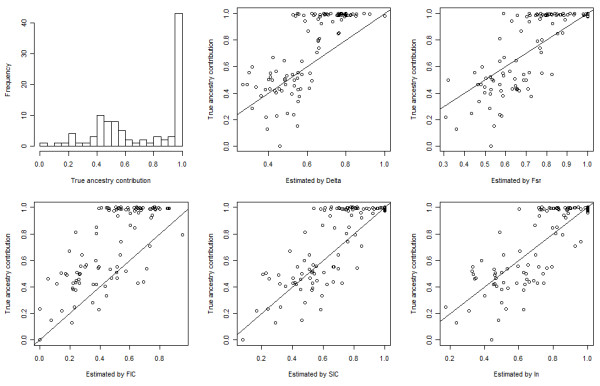
**Individual true ancestry contributions and estimated contributions using top 50 AIMs of the simulated admixed population from CHB and JPT**. Top-left panel: histogram of individual true ancestry contributions. Top-middle panel to bottom-right panel: scatter plot of individual true ancestry contributions vs. individual estimated contributions using top 50 AIMs selected by Delta, F_ST_, FIC, SIC, and I_n_, respectively.

**Figure 9:**
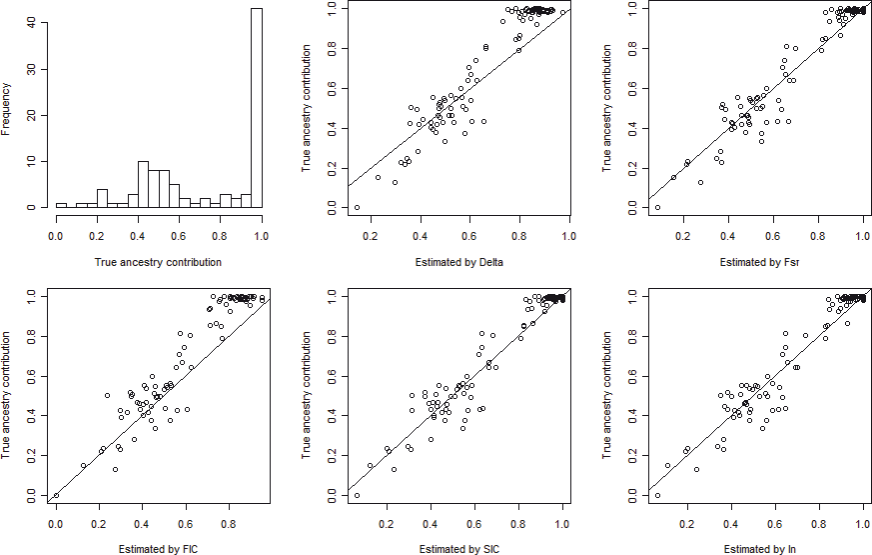
**Individual true ancestry contributions and estimated contributions using top 1000 AIMs of the simulated admixed population from CHB and JPT**. Top-left panel: histogram of individual true ancestry contributions. Top-middle panel to bottom-right panel: scatter plot of individual true ancestry contributions vs. individual estimated contributions using top 1000 AIMs selected by Delta, F_ST_, FIC, SIC, and I_n_, respectively.

For the simulated admixed population from CHB and JPT, a random sample of 100 individuals was extracted. The true average ancestry contribution was 72:28 for the simulated admixed population. Absolute errors in the estimation of the ancestry contribution for the simulated population with up to top 100 AIMs selected by different measures of informativeness are given in Additional file 9, Figure S6 (b). The bias in the estimation of mean ancestry contribution was generally higher than that for the simulated admixed population from CEU and YRI as shown in Additional file 9, Figure S6 (a). Specifically, the lowest bias of each method was 0.09 by δ with 100 AIMs, 0.005 by F_ST _with 50 AIMs, 0.15 by FIC with 20 AIMs, 0.07 by SIC with 50 AIMs, and 0.01 by I_n _with 50 AIMs. Using top 50 AIMs, RMSE's were 0.218, 0.182, 0.306, 0.186, and 0.170 for δ, F_ST_, FIC, SIC, and I_n_, respectively. Figure 8 shows the histogram of the individual true ancestry contributions and the scatter plot of individual estimated contributions vs. true contributions with 50 top AIMs selected by different methods. The individual estimates became more accurate as more AIMs were used (Figure 9). Overall, relative large true ancestry contributions were more likely to be underestimated by all measures. However, across the two simulation scenarios, I_n _gave the lowest bias and RMSE using only relatively small AIM panels.

#### Estimation of ancestral contribution in admixed populations with random subsets of top AIM panels

**Figure 10:**
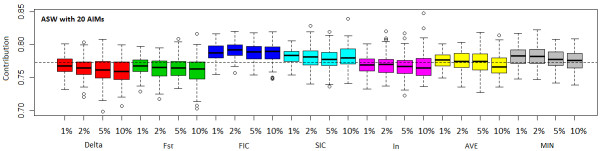
**Box-and-Whisker plot of estimates of mean ancestry contribution for ASW population with 100 random subsets of 20 SNPs from panels consisting of top 1%, 2%, 5%, and 10% of the AIMs**. From left to right different colors indicate results for Delta, F_ST_, FIC, SIC, and I_n_. The last two sets of the plot (yellow and gray) are the results where markers were ranked by the average rank (AVE) or minimum rank (MIN) of all five measures. The dashed line indicates 78%, which was estimated by a collection of 3299 AIMs for CEU and YRI population from HapMap phase III data. All of the 3299 markers were selected as top 10% AIMs by at least one of the five measures. For each method (color), the four Box-and-Whisker plots from left to right represent analysis results based on AIM panels consisting of top 1%, 2%, 5%, and 10% of the AIMs. See Additional File 10, Table S4 for summary statistics of estimation errors.

Figure 10 shows the ancestry estimation for the ASW population based on 100 sets of 20 randomly selected AIMs from the top 1%, 2%, 5%, and 10% AIMs of each measure of informativeness. Additional file 10, Table S4 shows summary statistics of errors in the estimation of population average ancestry contribution, where the 'true' or gold standard value, 78%, was estimated by a collection of 3299 AIMs for the CEU and YRI population, all of which were selected as top 10% AIMs by at least one of the five measures. As the analysis included more markers of less ancestry informativeness, the estimate of the YRI contribution tended to steer away from 78%, and the mean and the standard deviation of the errors across 100 simulations had an increasing trend for all the measures (Additional file 10, Table S4). AIMs chosen by I_n _gave the smallest mean error whereas those chosen by FIC had the highest mean error. AIMs chosen by FIC and SIC were more likely to overestimate ancestry contribution, and those by Delta, F_ST_, and I_n _were more likely to underestimate ancestry contribution.

**Figure 11:**
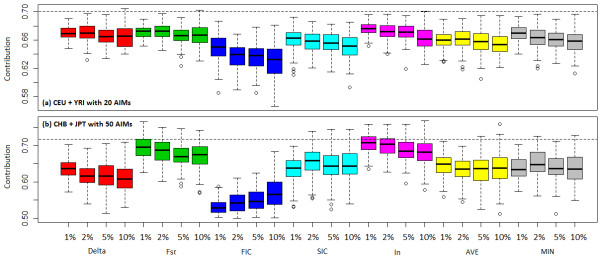
**Box-and-Whisker plot of estimates of mean ancestry contribution for the simulated admixed populations with 100 random subsets of 20 or 50 SNPs from panels consisting of top 1%, 2%, 5%, and 10% of the AIMs**. (a) Results for the simulated admixed population from CEU and YRI using random subsets of 20 AIMs. The dashed line indicates the true mean ancestry contribution (70%). (b) Results for the simulated admixed population from CHB and JPT using random subsets of 50 AIMs. The dashed line indicates the true mean ancestry contribution (72%). From left to right different colors indicate results for Delta, F_ST_, FIC, SIC, and I_n_. The last two sets of the plot (yellow and gray) are the results where markers were ranked by the average rank (AVE) or minimum rank (MIN) of all five measures. For each method (color), the four Box-and-Whisker plots from left to right represent analysis results based on AIM panels consisting of top 1%, 2%, 5%, and 10% of the AIMs. See Additional File 11, Table S5 and Additional File 12, Table S6 for summary statistics of estimation errors.

Results of simulation studies are shown in Figure 11(a) and Additional file 11, Table S5 for the admixed population from CEU and YRI, where random subsets of 20 AIMs were used, and Figure 11(b) and Additional file 12, Table S6 for the admixed population from CHB and JPT, where random subsets of 50 AIMs were used. From the results we make the following observations. First, even with less AIMs, the estimates for the admixed population from CEU and YRI were more accurate than those for the admixed population from CHB and JPT, which is expected since CEU and YRI are more divergent genetically than CHB and JPT. Secondly, for all the five measures, as the AIM panels included more markers that were less ancestry informative, the mean and standard deviation of estimation errors increased. Finally, AIM panels chosen by I_n _gave the smallest mean error, whereas those chosen by FIC gave the largest mean error. The superiority of I_n _was evident in the simulation study using CHB and JPT (Additional file 12, Table S6). However, different from what we observed in the ASW population, FIC and SIC underestimated ancestry contribution. A possible interpretation for the observed discrepancy between the ASW data and the simulated data is that FIC and SIC are more sensitive to the underlying distribution of the ancestry contribution of individuals in the admixed population. The other possibility could be that the assumption we made about the genetic structure of ASW population is wrong. Although we tried to mimic the formulation of an African-American population in our simulation (assuming only two ancestral populations involved and have been correctly specified), the simulated data was more than likely to be different from the true African-American population. Simulated and empirical data differ in that the simulations used fully-differentiated populations, which is not the case for the empirical data. The 3D scatter plot of CEU and YRI allele frequencies and the five measures of informativeness in Figure 2 also showed the asymmetry of FIC and SIC, which may contribute to their sensitivity to the underlying distribution of individual ancestry contribution.

Overall, the AIM panels chosen by I_n _performed the best, giving the lowest bias and RMSE, whereas those by FIC performed the worst across the real dataset and the simulated datasets. For the real dataset (ASW), the combined method by using the average ranking of the five measures outperformed all the five measures (Additional file 10, Table S4). However, for the simulated datasets, the average ranking and minimal ranking methods were outperformed by I_n _and F_ST_, but their performance were either better or very similar to the most commonly used method Delta, FIC and SIC (Additional file 11, Table S5 and Additional file 12, Table S6).

## Discussion

Admixture mapping is a powerful gene mapping approach [5,28]. However, the power of this method relies on the ability of ancestry informative markers (AIMs) to infer ancestry along the chromosomes of previously separated but recently admixed individuals. In a recent paper from ASHG, Royal *et al*. (2010) [29] outlined the challenges, opportunities and implications of genetic ancestry inference. Over 40 companies provide genetic ancestry testing to the public. However, these companies differ in their approaches, the types of ancestry markers used and tests they offer. The promise of utilizing genetic ancestry information to advance medical genomics depends on our ability to correctly and precisely infer/measure ancestry using informative markers.

Several methods have been proposed to measure ancestry informativeness of markers and to choose a panel of markers to be genotyped while maintaining the power of detecting ancestral chromosome segments in each genomic location. The choice of which of these measures to use should depend on the efficiency of each measure in selecting most ancestry informative markers. However, there is no consensus as to which criteria to use to select markers for ancestry inference or admixture mapping, and the performance of these methods has not been carefully evaluated and compared. The rule of thumb is to select markers with large allele frequency differences between ancestry populations. However, the number of markers required for population assignment will depend on the populations under consideration, their respective level of genetic differentiation and the desired stringency of assignment [30]. For instance, in humans, the level of genetic variation between populations is only 5%-10% whereas genetic variation within dogs is about 27% [31]. As a result, the number and types of markers required for individual assignment and discrimination amongst populations is different between populations/species under consideration [30]. Previous studies selected markers based on different datasets and marker types using only one of the methods at a time, and there has not yet been a formal comparison of the performance of these methods. Therefore there is a need to compare all methods using the same data sets and evaluate their efficiencies and accuracies in estimating ancestral proportions for admixed populations.

In this study, we applied five different analytic tools to evaluate the concordance of selected informative SNPs using the same dataset. Our investigation using 500 top ranked markers for each measure and accounting for the physical distance between consecutive AIMs to be at least 100 kb, showed the following overlap between the different measures: δ vs F_ST _(n = 479), δ vs FIC (n = 220), δ vs SIC (n = 319), δ vs I_n _(n = 424), F_ST _vs FIC (n = 230), F_ST _vs SIC (n = 329), F_ST _vs I_n _(n = 445), FIC vs SIC (n = 395), FIC vs I_n _(n = 258), and SIC vs I_n _(n = 354) (Additional file 13, Table S7). On average, the overlap of each measure with the other four was 361, 371, 276, 349, and 370 for δ, F_ST_, FIC, SIC, and I_n_, respectively. FIC had the least overlap with other measures. However, based on current cutoff values used for each measure, the δ measure included a number of loci that were not selected by the remaining four methods. F_ST_, FIC, and I_n _gave relatively smaller and similar AIM panels, whereas SIC gave a very small panel of AIMs (Additional file 14, Figure S7). Analyses based on deciles showed that sets of SNPs at the highest or lowest SNP information content selected for admixture mapping were highly similar across the different measures of informativeness. Towards the middle of the informativeness scales, the agreement among the sets of SNPs selected by different methods to discriminate between populations decreased (Figure 3, 4, and 5). Furthermore, FIC and SIC were more likely to pick the same set of SNPs, δ, F_ST_, and I_n _were more likely to pick the same set of SNPs, and FIC was more likely to choose SNPs that were not chosen by the other measure.

Analytically, the FIC and SIC measures require pre-defined ancestral proportions in an admixed population, whereas F_ST_, δ, and I_n _do not. We ran sensitivity analysis to study the impact of ancestral proportion in choosing informative markers using arbitrary values using CEU and YRI population. Compared with FIC, SIC was less sensitive to the proportion of ancestry contribution in the selection of AIMs (Additional file 15, Table S8 and Additional file 16, Table S9). Proportion of ancestry had virtually no effect on the selection of top 1% AIMs for SIC. For two proportions of ancestry contribution within a distance of 0.1, FIC selected 56%-71% common sets of AIMs and SIC selected 75%-100% common sets of AIMs. Therefore, it is important, when using FIC, to have a good *a priori *estimate of proportion of ancestry contribution.

There are some limitations for some of these measures, for example, FIC favors selection of markers that are closer to fixation in one parental population and may not be appropriate to assess the level of informative markers when ancestral populations are more than two [32]. Compared with other methods such as F_ST_, δ is easy to calculate and independent of mutation and model assumptions, however, δ has a major limitation of being only useful for admixed populations from two parental populations and it doesn't account for multiallelic situations at a locus. F_ST _may not be appropriate to assess the level of genetic information in SNP markers when the number of populations is > 2, as the method could result in the selection of SNP markers which are specific for a single most genetically distinct population. The selected SNP markers that were specific for only the most distinct population are expected to have low heterozygosity. Genetic markers with high expected heterozygosity are informative and therefore useful in individual assignment analysis [33]. Although most researchers traditionally focus on global axes of variation in a dataset, substantial information about population ancestry exists locally- across chromosomes. Adjustment of global ancestry between study subjects may lead to false positives when chromosomal (local) population ancestry is an important confounding factor [34]. In a recent chromosome-based study by Baye (2011) [35], fine-scale substructure was detectable beyond the broad population level classifications that previously have been explored using genome-wide average estimates. The study of population ancestry in terms of local ancestry has broader practical relevance because genetic diversity is directly related to recombination rate (meiosis), which differs among regions of the genome, and genes are not randomly distributed along chromosomes. The current analytical approach using genome-wide average estimates will control for confounding due to global ancestry but will not control for confounding due to the local ancestry effect because the global ancestry information is obtained from all markers across the genome and may not accurately reflect local ancestry variation. It is becoming increasingly important to recognize local ancestry variation, especially when populations have been recently admixed [35]. Future studies should focus on the applications of these measures to important genomic regions.

Many factors impact the accuracy of the estimation of ancestry contributions, which include but are not limited to sample size, the panel of AIMs used, the number of AIMs used, and the underlying distribution of ancestry contribution of the individuals in the sample. The use of a phased HapMap dataset allowed us to simulate individuals that share common founding populations. Moreover, the ancestry proportion for each individual is known, allowing for the comparison of true and estimated individual admixture values, thus enabling the comparison of different methods by estimation accuracy. Our findings indicate that, the different measures of marker informativeness [δ, F_ST_, FIC, SIC, and I_n_] performed well and as few as the top 20 ranked informative markers were adequate for accurate classification of ancestral populations. This is in agreement with the commonly made claim in the literature on marker selection for population assignment that 'classification accuracy can be substantially improved if only a subset of loci is used in the assignment test' [36]. For instance, Lao et al., (2006) [37] found that 10 SNP markers from a 10 K SNP array contained enough genetic information to differentiate individuals from Africa, Europe, Asia and America and no further gain in power of assignment was achieved by including more SNP markers. Indeed, it is generally considered that uninformative markers (i.e., monomorphic loci) may add variability and noise to the results and compromise the power of population genetic studies [38].

Although the marker selection methods explored in this study agreed to a large extent in identifying the most informative SNPs, there were differences in their performance in ancestry estimation. The simulation study revealed that I_n _was the best in selecting the set of AIMs giving the smallest bias and mean square error in ancestry estimation. Analysis based on random subsets of top 1% to 10% ranked AIMs indicated that, compared to other methods, AIM panels selected by I_n _behaved consistently and reasonably well for both the ASW population and simulated admixed populations. These results illustrate that effective exploration of all these methods can help to not only identify the most informative markers but also produce an optimal minimum set of markers that can accurately and efficiently differentiate among populations.

We suggest that the different measures may provide unique insights into a marker's informativeness under different scenarios, including varying ancestral proportion and when more than two ancestral populations are present. To identify all potentially informative SNPs, results from all measures could be considered. For example, the union of the top 500 SNPs for all five measures could be considered as the best AIMs panel. Researchers need to be aware of the differences between the various methods for evaluating ancestry informativeness of SNP markers. Furthermore, as we attempted in our simulation studies using either average rank or minimal rank of all five measures, combined information from more than one method may provide a reliable means, although may not be the best, in selecting markers for ancestry inference. Further research on this topic may shed light on how to best integrate different measures to obtain a set of AIMs most effective for the populations under consideration. We believe that the information that a set of markers provides for assigning or discriminating individuals to their source populations or different relationships must be critically evaluated before investing millions of dollars on an admixture or ancestry related project. We anticipate identification of more complex patterns of ancestry will require explorations of these and newer methods yet to be defined, to identify an optimal set of markers to use, however this should become increasingly feasible as genotyping costs decrease and available data grow on different populations. This in turn will allow the development of higher resolution of genogeographic and ethnic maps and help investigators designing genetic association studies in stratified homogeneous groups.

## Conclusions

Although millions of SNP markers with varying levels of information content for ancestry inference have been identified, only small subsets of highly informative markers need to be genotyped in order to accurately predict ancestry with a minimal error rate in a cost-effective manner. In this article, we compared various methods for selecting informative SNPs and showed that the I_n _measure estimated ancestry proportion (in an admixed population) with lower bias and mean square error. In summary, we showed the utility of several measures of informativeness using simulations and real SNP genotype data from samples of admixed populations. The use of several available methods to prioritize informative markers for ancestry inference can reduce genotyping costs and avoid false positive genotype-phenotype associations while retaining most of the power found in much larger sets of published AIM panels.

## Methods

### Five measures of ancestry informativeness

#### Notation

Consider populations *i *= 1, 2,..., *K *with *K *≥ 2 and a locus with *N *≥ 2 alleles. Let *p*_*ij *_denote the frequency of allele *j, j *= 1, 2, ..., *N*, in population *i*. Let *p*_*j *_denote the average frequency of allele *j *over the *K *populations, i.e., $p_{j}=\sum_{i=1}^{K}p_{ij}∕K$. Consider an admixed population with two parental populations, the frequency of allele *j *at a locus in the admixed population *p*_*Aj *_is a linear combination of allele frequencies in the ancestral populations, and can be written as *p*_*Aj *_= *m*_1_*p*_1*j *_+ *m*_2_*p*_2*j*_, where *m*_*i *_is the proportion of contribution of the *i*^th ^ancestral population, and *m*_1 _+ *m*_2 _= 1.

#### Absolute allele frequency difference (delta, δ)

Delta is the most commonly used measure of SNP marker informativeness for ancestry between two parental populations. Delta is defined as the absolute difference in the frequencies of a particular allele observed in two ancestral populations. For a biallelic locus, suppose allele one is the reference allele, then,

$$\delta =\left|p_{11}-p_{21}\right|.$$

A marker with δ = 1 provides perfect information regarding ancestry whereas a marker with δ = 0 carries no information. It has been shown that δ by itself only provides limited information regarding a marker's informativeness for ancestry [10]. The sum of the allele frequencies in the two parental populations, or equivalently the value of the smaller of the two frequencies, can provide additional information independent of δ.

#### F statistics (F_ST_)

F_ST _is the proportion of the total genetic variance (the T subscript) [39] contained in a subpopulation (the S subscript). When only two parental populations and markers with only two alleles are considered, the informativeness for ancestry includes the differences and the sum of the reference allele frequencies in the two parental populations. In other words,

$$F_{ST}=\frac{{\left(p_{1j}-p_{2j}\right)}^{2}}{\left(p_{1j}+p_{2j}\right)\left(2-\left(p_{1j}+p_{2j}\right)\right)}.$$

Here, *j *= 1 or 2 is the reference allele. Values of F_ST _can range from 0 to 1. A high F_ST _value implies a considerable degree of differentiation between populations. F_ST _is a pair-wise population measure of differentiation or relatedness (genetic distance measure between the two populations) based on genetic polymorphism data such as SNPs and was recently utilized as a criterion for selecting markers for ancestry estimation [40].

#### Fisher Information Content (FIC)

Pffaff [41] showed how FIC can be used to determine the informativeness of a specific marker. The determinant of the Fisher information matrix provides a measure of the amount of information contained in the data. The genetic contributions of the ancestral populations can be estimated by the maximum likelihood method from a sample of genotypes from the admixed population [42,43]. For a biallelic locus of an admixed population from two parental populations,

$$FIC=\overset{2}{\underset{j=1}{\sum }}\frac{{\delta_{j}}^{2}}{{\hat{p}}_{Aj}}.$$

Here, ${\hat{p}}_{Aj}=p_{2j}+{\hat{m}}_{1}\delta_{j}$ is the expected frequency of the *j*^th ^allele in the admixed population or individual, *δ*_*j *_= *p*_1*j *_- *p*_2*j *_is the allele frequency difference, ${\hat{m}}_{1}$ is the maximum likelihood estimate of the contribution from ancestral population one. FIC measure allows selection of markers that are particularly informative in an admixed population in which the contribution of one parental population is substantially greater than that of the other parental population. It favors selection of markers that are closer to fixation in the parental population with the greater contribution.

#### Shannon Information Content (SIC)

Rosenberg et al. (2003) [10] used the concept of entropy to develop a measure of marker informativeness. Entropy is a measure of the uncertainty associated with a random variable and quantifies the expected information content contained in the data. If the sampled population is an admixture of two parental populations (with ancestral proportion m_1 _and m_2_), the SIC for a biallelic locus can be written as:

SIC=m1 ∑j=12p1j logp1j+m2 ∑j=12p2j logp2j-∑j=12pAj logpAj.

#### Informativeness for assignment (I_n_) measure

I_n _is a mutual information-based statistics that takes into account self-reported ancestry information from the sampled individuals [10]. The informativeness for assignment of a SNP is defined as:

$$I_{n}=\overset{N}{\underset{j=1}{\sum }}(-p_{j}\log_{2}p_{j}+\overset{K}{\underset{i=1}{\sum }}\frac{p_{ij}\log_{2}p_{ij}}{K}).$$

This formula is a generalization to more than two populations. From a likelihood perspective, it gives the expected logarithm of the likelihood ratio that an allele is assigned to one of the populations compared with a hypothetical 'average' population whose allele frequencies equal the mean allele frequency across the *K *populations. Its value is smaller when all alleles have similar frequencies in all populations.

### Data

#### HapMap phase III dataset

We downloaded the complete HapMap phase III genotype data (http://www.hapmap.org, release #3, May 2010) available for Yoruban population in Ibadan, Nigeria [YRI], Caucasian population from the United States with northern and western European ancestry [CEU], and the African American population from Southwest USA [ASW]. HapMap is a public resource created by the International HapMap Project to catalogue genetic variants (SNPs) that are common in human populations. The HapMap phase III release #3 contains genotypes from 147 unrelated individuals (parents) from YRI population, 113 unrelated parents from CEU population, and 87 individuals from ASW population. For the purpose of the present study, YRI and CEU populations are assumed to be the ancestors of ASW population. Two criteria were used to filter the SNPs included in the final analysis: 1) the SNP should be shared by both YRI and CEU populations, i.e., SNPs for which allele frequencies were available in both YRI and CEU populations, and 2) SNPs with missing frequency for over 10% of the samples were excluded. Furthermore, to avoid the possibility of choosing two redundant SNPs that are in strong LD (linkage disequilibrium), for each measure, we calculated the informativeness on all shared SNPs, then filtered them for the most informative ones such that the physical distance between consecutive selected SNPs must be at least 100 kb.

#### Simulated dataset

To compare marker informativeness measures in the estimation of ancestry population contribution, we simulated two artificially admixed datasets from the phased HapMap III dataset (with known allele frequencies). The first one is an admixed population from two parental populations with relatively high divergence: 113 unrelated individuals in CEU population and 113 unrelated individuals in YRI population. The second one is an admixed population from less differentiated ancestral populations: 84 unrelated individuals of Han Chinese in Beijing, China [CHB] and 86 unrelated individuals of Japanese in Tokyo, Japan [JPT]. The simulations were run using simuPOP [32,33] for 10 generations. During the simulation, we tracked the true ancestry contributions for each individual and calculated average ancestry contributions for each of the two admixed populations.

### Statistical analysis

Python (http://www.python.org) scripts were written to retrieve and pre-process SNP and frequency data. Five measures of marker ancestry informativeness were calculated for shared SNPs between CEU and YRI population and for shared SNPs between CHB and JPT population, with YRI or JPT contribution fixed as 80% for the calculation of FIC and SIC. Sensitivity analysis of different YRI contributions on the selection of AIMs was performed for FIC and SIC. For each data set, the number of alleles per locus (SNP) was coded to a string of numbers to obtain a full design matrix of alleles where the cells give the number of copies of each major allele for each individual (zero, one, or two). R (R Foundation for Statistical Computing, 2010), SAS software (SAS 9.1.3, SAS Institute Inc.), and JMP Genomics (JMP^® ^Genomics, v.5, SAS Institute Inc.) programs were used to analyze the various measures of informativeness for ancestry.

### Correlation, concordance, and overlapping analysis

To assess the level of similarity of the estimates of genetic information contained in each SNP marker across the five measures of marker informativeness, we used three statistical procedures: Spearman correlation coefficient, Cohen's Kappa statistics, and overlapping frequency analysis of top *n *ranked AIMs by different measures. Although the three approaches share some common information, each provides unique and complementary views of the behavior of the five measures. Spearman correlation coefficient is a global measure of statistical dependence and provides a general sense regarding pair-wise monotonic relationship of the five measures of marker informativeness. The Cohen's Kappa coefficient based on deciles quantifies agreement between two measures and the corresponding mosaic plot exhibits overlapping structures, i.e., the distribution of markers according to one measure of informativeness relative to another. Finally, the overlapping frequency analysis demonstrates how often the same set of SNPs is selected by two or more different measures or which measures tend to select the same set of SNPs.

Spearman correlation coefficient [44] is a measure of correlation based on the ranks of the data values. It is a nonparametric alternative to Pearson's correlation coefficient and do not require the knowledge of the distribution of the data. The formula for Spearman correlation coefficient is $\frac{\sum_{i}\left(\left(X_{i}-\bar{X}\right)\left(Y_{i}-\bar{Y}\right)\right)}{\sqrt{\sum_{i}{\left(X_{i}-\bar{X}\right)}^{2}\sum_{i}{\left(Y_{i}-\bar{Y}\right)}^{2}}}$, where, *X*_i _and *Y*_i _are the ranks of observed data values, $\bar{X}$ is the mean of *X*_i_'s, and $\bar{Y}$ is the mean of *Y*_i_'s. In case of ties, the averaged ranks are used. Spearman correlation coefficient takes values between -1 and +1. A +1 or -1 indicates that the two measures are in a perfectly monotonically increasing or decreasing relationship, respectively, and a 0 means no relationship.

To show the distribution of markers according to one measure of informativeness relative to another, we further analyzed the data by grouping and rating SNP markers using deciles, producing mosaic plots and calculating Cohen's Kappa coefficients. Deciles are the nine values of a variable dividing its distribution into ten groups with equal frequencies. For each measure, based on its deciles we created a new categorical variable with values 1, 2..., and 10, indicating to which group a SNP belongs. We then used the new categorical variables to build mosaic plots and to examine the relationship between measures of marker informativeness. The mosaic plots show, for example, how the top 10% SNPs from one measure of informativeness distribute relative to another measure of informativeness. To assess the concordance of decile-based ratings of the informativeness of AIMs between measures, we computed the Cohen's kappa coefficient, a commonly used index to quantify agreement between two measurements [45]. It takes into account the concordance by chance and is calculated as κ = [Pr(*a*) - Pr(*e*)]/[1- Pr(*e*)], where Pr(*a*) is the observed agreement percentage, and Pr(*e*) is the chance agreement percentage. The larger the kappa coefficient, the better the concordance is between two measurements. Kappa takes values between 0 and 1. *κ = *1 indicates a perfect agreement while κ = 0 indicates no agreement other than what would be expected by chance.

To answer the question of how often the same set of SNPs is selected by the different methods or which methods tend to select the same set of SNPs, we studied the overlap pattern of the top *n *AIMs selected by different measures of informativeness. Each SNP was assigned a 5-digit binary vector, where each digit represents a measure. From the first to the last these correspond to δ, F_ST_, FIC, SIC, and I_n_, respectively. A 1 in the digit indicates that the SNP is selected by the corresponding measure as one of the top *n *AIMs. For example, a binary vector 11001 represents the SNP is selected by δ, F_ST_, and I_n _as one of the top *n *AIMs, but not by FIC and SIC. For a specific *n*, the frequency of the different combinations of the 5-digit numbers (such as 11001 and 00110) shows how often the different methods select the same set of SNPs. The higher the frequencies, the higher the chance that the same set of SNPs are selected by the methods corresponding to the 1's in the 5-digit vector.

### Discriminant analysis

To compare the discrimination power of the five measures of informativeness and assess how many markers are needed for accurate ancestral CEU vs. YRI population and CHB vs. JPT population membership assignment, discriminant analysis was performed using the top 1, 2, ..., and up to 150 ranked AIMs. Discriminant analysis [46] is a method of projecting high-dimensional data onto a lower-dimensional space in a way that data points from different classes are well-separated. The projection is given by $y=\sum_{i=1}^{p}w_{i}x_{i}$, where *x *= (*x*_1_, *x*_2_, ..., *x*_*p*_) is a *p *dimensional data point (individuals with SNP information), and *y *is the projection of *x *onto *w *= (*w*_1_, *w*_2_, ..., *w*_*p*_), or a linear combination of *x*_*i *_with weights *w*_*i*_. The weights are chosen such that the projections of the data points (individuals) in the same class (CEU or YRI population) are close to each other while those of the data points from different classes are far from each other. Linear discriminant can be derived using a measure of generalized squared distance. An optimal linear classifier then can be found by minimizing classification error (probability of misclassification). The classifier can take into account of prior probabilities of the classes, which, in our analysis, were specified as proportional to the sample sizes in each class. A data point is classified into the class for which the posterior probability of the observation belonging to this class is the largest among all classes. Cross-validation is used to obtain prediction accuracy. The analysis was carried out using PROC DISCRIM in SAS (SAS 9.1.3, SAS Institute Inc.). We also examined the number of AIMs needed to achieve 90% or 95% classification accuracy.

### Estimation of ancestry contribution in admixed ASW population

We estimated ancestry contribution for the admixed ASW population using up to 200 top ranked AIMs by different measures. We also estimated ancestry contribution using 100 sets of randomly selected 20 SNPs from the top 1%, 2%, 5%, and 10% ranked AIM panels. The analysis was performed using the software PSMIX [47]. This analysis allowed us to compare the consistency in the estimation of ancestry contribution when the number of informative markers in the pool increases or decreases. The rationale for conducting this analysis is that AIM panels generated by different measures are more similar when only the top AIMs are considered, which makes it difficult to compare their performances using only the top AIMs. By using random subsets of AIMs from the top AIM panels of various sizes, we expect to select less informative markers and the estimate of the ancestry contribution is expected to become less accurate (or more biased) with more variability. More importantly, we will be able to determine if there is clear separation in the performance of different measures based on the information content taken from similar (1% to 10%) pools of markers as determined by each method.

We constructed two new methods of ranking marker informativeness for ancestry by combining the information from all the five measures. For each marker, we assigned a ranking or score based on either the average ranking (AVE) or the minimum ranking (MIN) of the five measures. We didn't use the raw values from the five measures because they have different scales; thus, any score computed by weighted average of the raw values needs to be preceded by standardization of the raw values, which is beyond the scope of this paper.

### Estimation of ancestry contribution in simulated admixed population

To validate the ancestral estimates of the five measures, the same set of analyses in the previous section were conducted for the two simulated admixed populations. In the simulated admixed populations, the ancestry proportion for each individual is known, so is the mean ancestry proportion across individuals in the same population. Estimation accuracy by different measures was compared at two different levels. At the population level, the estimate of the mean ancestry contribution across individuals was compared with the true value and bias was calculated for the five measures. At the individual level, individual true and estimated admixture values were compared, and root mean square error (RMSE) was used as a summary measure of precision in the estimation of individual ancestry proportion. RMSE is defined as ${\left[\frac{1}{M}\overset{M}{\underset{i=1}{\sum }}{\left(q_{i}-{\hat{q}}_{i}\right)}^{2}\right]}^{1∕2}$, where *M *is the number of individuals in the sample, and *q*_*i *_and ${\hat{q}}_{i}$ represent the true and estimated individual ancestries, respectively. We also plotted individual estimated contributions vs. true contributions.

## Abbreviations

AIMs: ancestry informative markers; GWAS: genome-wide association studies; YRI: Yoruban population in Ibadan, Nigeria; CEU: Caucasian population from the United States with northern and western European ancestry; ASW: African American population from Southwest USA; CHB: Chinese population from Beijing, China; JPT: Japanese population from Tokyo, Japan.

## Competing interests

The authors declare that they have no competing interests.

## Authors' contributions

TMB conceived of the study and drafted the manuscript. LD performed the data analysis and manuscript writing. HW, TA, MA, RCPG, CK, LM, GKKH and RC contributed in manuscript writing. All authors read and approved the final manuscript.

## Web resources

HapMap: http://www.hapmap.org

PYTHON: http://www.python.org

## Supplementary Material

Additional file 1 **Table S1: Summary statistics of five measures of marker informativeness for CEU and YRI population in the HapMap phase III data**. A table of mean, standard deviation, minimum, median, maximum, and lower and upper quartile of the five measures of marker informativeness for CEU and YRI population. Click here for file

Additional file 2 **Figure S1: Distribution of the five measures of marker informativeness for CHB and JPT population from HapMap phase III data**. Histograms of the five measures of marker informativeness. Almost all the SNP markers displayed low estimates of genetic informativeness. Click here for file

Additional file 3 **Table S2: Summary statistics of five measures of marker informativeness for CHB and JPT population in the HapMap phase III data**. A table of mean, standard deviation, minimum, median, maximum, and lower and upper quartile of the five measures of marker informativeness for CHB and JPT population. Click here for file

Additional file 4 **Table S3: Kappa statistics of the five measures of informativeness as defined by deciles**. A table of pair-wise Kappa statistics of the five measures of informativeness. Click here for file

Additional file 5 **Figure S2: Scatter plot of allele frequencies of CEU and YRI population partitioned by the ten groups defined by deciles of each measure of informativeness**. The top-left and bottom-right corner represent the most informative SNPs whereas the least informative SNPs reside at the center of the plot. Click here for file

Additional file 6 **Figure S3: Number of AIMs needed to achieve specific accuracies for founder populations**. The two founder populations are (a) CEU and YRI and (b) CHB and JPT. Click here for file

Additional file 7 **Figure S4: Inferred population structure for CEU, YRI and ASW population with two clusters and 200 AIMs selected by FIC**. A plot of the inferred population structure of CEU, YRI and ASW population. The analysis was done in STRUCTURE and *distruct *with 2 clusters. Click here for file

Additional file 8 **Figure S5: Estimate of ancestry contribution vs. number of top AIMs for CEU, YRI and ASW population from HapMap phase III data**. Top panel: estimate of CEU contribution for CEU population. Middle panel: estimate of YRI contribution for YRI population. Bottom panel: estimate of YRI contribution for ASW population. Click here for file

Additional file 9 **Figure S6: Absolute error in the estimation of mean ancestry contribution for the simulated admixed populations**. A plot of absolute error in the admixed population simulated from (a) CEU and YRI and (b) CHB and JPT. Click here for file

Additional file 10 **Table S4: Summary statistics of estimation errors of mean ancestry contribution for ASW population**. The estimates were based on 100 random subsets of 20 SNPs from panels consisting of top 1%, 2%, 5%, and 10% of the AIMs for CEU and YRI population. The gold-standard or 'true' ancestry contribution was taken as 78%, estimated by a collection of 3299 AIMs for the CEU and YRI population, all of which were selected as top 10% AIMs by at least one of the five measures. Click here for file

Additional file 11 **Table S5: Summary statistics of estimation errors of mean ancestry contribution for the simulated admixed population from CEU and YRI**. The estimates were based on 100 random subsets of 20 SNPs from panels consisting of top 1%, 2%, 5%, and 10% of the AIMs for CEU and YRI population. The true ancestry contribution was 70%. Click here for file

Additional file 12 **Table S6: Summary statistics of estimation errors of mean ancestry contribution for the simulated admixed population from CHB and JPT**. The estimates were based on 100 random subsets of 50 SNPs from panels consisting of top 1%, 2%, 5%, and 10% of the AIMs for CEU and YRI population. The true ancestry contribution was 72%. Click here for file

Additional file 13 **Table S7: Overlap of SNP markers between measures**. Diagonal (bolded): Number of SNPs genotyped in both populations and satisfying the filtering criteria^a^. Upper-triangle: Overlap for the SNP markers. Lower-triangle: Overlap for the top 500 ranked SNP markers. Click here for file

Additional file 14 **Figure S7: Scatter plot of allele frequency difference between CEU and YRI population using current cutoff values for each measure**. Markers in red exceeded the cutoff for the measure of informativeness. Similar patterns were observed between F_ST _and I_n_. Delta yielded the largest AIMs panel and included a large number of loci not included by any of the remaining four methods. SIC gave the smallest AIMs panel. Click here for file

Additional file 15 **Table S8: FIC - Sensitivity analysis of proportion of ancestry contribution on the selection of AIMs**. For a pair of proportions of ancestry contribution (m and m'), we examined overlap patterns between the two top n% AIM panels selected using m and m' in the computation of FIC. Overlap patterns were presented by 11: AIMs selected by both panels; 10: AIMs selected by panel one (m) but not panel two (m'); and 01: AIMs selected by panel two (m') but not panel one (m). Frequency and percentage of each overlap pattern were reported for top 1%, 5%, 10%, and 20% AIMs. Proportion of ancestry contribution considered included 0.1, 0.2, 0.3, 0.4, and 0.5. Click here for file

Additional file 16 **Table S9: SIC - Sensitivity analysis of proportion of ancestry contribution on the selection of AIMs**. For a pair of proportions of ancestry contribution (m and m'), we examined overlap patterns between the two top n% AIM panels selected using m and m' in the computation of SIC. Overlap patterns were presented by 11: AIMs selected by both panels; 10: AIMs selected by panel one (m) but not panel two (m'); and 01: AIMs selected by panel two (m') but not panel one (m). Frequency and percentage of each overlap pattern were reported for top 1%, 5%, 10%, and 20% AIMs. Proportion of ancestry contribution considered included 0.1, 0.2, 0.3, 0.4, and 0.5. Click here for file
