# Supplementary material for: Comparison of measures of marker informativeness for ancestry and admixture mapping
Source: BMC Genomics. 2011 Dec 20;12:622. doi: 10.1186/1471-2164-12-622 (PMC3276602; doi:10.1186/1471-2164-12-622)
Supplement: Additional file 1 — Table S1: Summary statistics of five measures of marker informativeness for CEU and YRI population in the HapMap phase III data. A table of mean, standard deviation, minimum, median, maximum, and lower and upper quartile of the five measures of marker informativeness for CEU and YRI population. [file 1471-2164-12-622-S1.DOCX]

**Additional file 1**

**Table S1: Summary statistics of five measures of marker informativeness for CEU and YRI population in the HapMap phase III data**

| Measure | Mean | Std Dev | Min | Median | Max | Lower | Upper |
| --- | --- | --- | --- | --- | --- | --- | --- |
|  |  |  |  |  |  | Quartile | Quartile |
| Delta F_ST_ FIC SIC I_n_ | 0.1890 0.0744 0.3513 0.0269 0.0585 | 0.1512 0.0939 0.4996 0.0360 0.0763 | 0 0 0 ~0 ~0 | 0.1523 0.0391 0.1586 0.0131 0.0294 | 0.9775 0.9556 5.7780 0.4451 0.9115 | 0.0693 0.0093 0.0373 0.0030 0.0068 | 0.2744 0.1036 0.4526 0.0364 0.0806 |
