# Supplementary material for: Comparison of measures of marker informativeness for ancestry and admixture mapping
Source: BMC Genomics. 2011 Dec 20;12:622. doi: 10.1186/1471-2164-12-622 (PMC3276602; doi:10.1186/1471-2164-12-622)
Supplement: Additional file 2 — Figure S1: Distribution of the five measures of marker informativeness for CHB and JPT population from HapMap phase III data. Histograms of the five measures of marker informativeness. Almost all the SNP markers displayed low estimates of genetic informativeness. [file 1471-2164-12-622-S2.DOCX]

**Additional file 2**

**Figure S1: Distribution of the five measures of marker informativeness for CHB and JPT population from HapMap phase III data.**

**
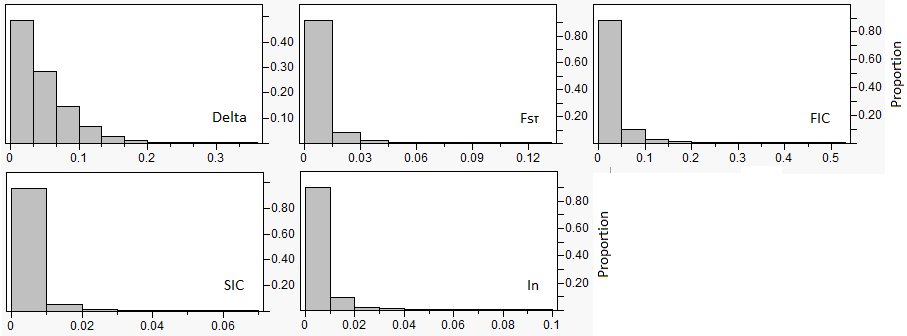
**

Almost all the SNP markers displayed low estimates of genetic informativeness.
