# Supplementary material for: Comparison of measures of marker informativeness for ancestry and admixture mapping
Source: BMC Genomics. 2011 Dec 20;12:622. doi: 10.1186/1471-2164-12-622 (PMC3276602; doi:10.1186/1471-2164-12-622)
Supplement: Additional file 3 — Table S2: Summary statistics of five measures of marker informativeness for CHB and JPT population in the HapMap phase III data. A table of mean, standard deviation, minimum, median, maximum, and lower and upper quartile of the five measures of marker informativeness for CHB and JPT population. [file 1471-2164-12-622-S3.DOCX]

**Additional file 3**

**Table S2: Summary statistics of five measures of marker informativeness for CHB and JPT population in the HapMap phase III data**

| Measure | Mean | Std Dev | Min | Median | Max | Lower | Upper |
| --- | --- | --- | --- | --- | --- | --- | --- |
|  |  |  |  |  |  | Quartile | Quartile |
| Delta | 0.044988 | 0.038378 | 0 | 0.035043 | 0.356734 | 0.015051 | 0.065015 |
| FST | 0.005304 | 0.007511 | 0 | 0.002416 | 0.132025 | 0.000523 | 0.007062 |
| FIC | 0.021217 | 0.030044 | 0 | 0.009663 | 0.528102 | 0.002094 | 0.028248 |
| SIC | 0.002696 | 0.003865 | ~0 | 0.001209 | 0.067873 | 0.000262 | 0.003578 |
| In | 0.003890 | 0.005577 | ~0 | 0.001745 | 0.097920 | 0.000378 | 0.005162 |
