# Supplementary material for: Comparison of measures of marker informativeness for ancestry and admixture mapping
Source: BMC Genomics. 2011 Dec 20;12:622. doi: 10.1186/1471-2164-12-622 (PMC3276602; doi:10.1186/1471-2164-12-622)
Supplement: Additional file 4 — Table S3: Kappa statistics of the five measures of informativeness as defined by deciles. A table of pair-wise Kappa statistics of the five measures of informativeness. [file 1471-2164-12-622-S4.DOCX]

**Additional file 4**

**Table S3: Kappa statistics of the five measures of informativeness defined by deciles.**

|  | F_ST_ | FIC | SIC | I_n_ |
| --- | --- | --- | --- | --- |
| Delta | 0.47 | 0.42 | 0.43 | 0.43 |
| F_ST_ |  | 0.65 | 0.85 | 0.93 |
| FIC |  |  | 0.76 | 0.65 |
| SIC |  |  |  | 0.86 |
