# Supplementary material for: Comparison of measures of marker informativeness for ancestry and admixture mapping
Source: BMC Genomics. 2011 Dec 20;12:622. doi: 10.1186/1471-2164-12-622 (PMC3276602; doi:10.1186/1471-2164-12-622)
Supplement: Additional file 5 — Figure S2: Scatter plot of allele frequencies of CEU and YRI population partitioned by the ten groups defined by deciles of each measure of informativeness. The top-left and bottom-right corner represent the most informative SNPs whereas the least informative SNPs reside at the center of the plot. [file 1471-2164-12-622-S5.DOCX]

**Additional file 5**

**Figure S2: Scatter plot of allele frequencies of CEU and YRI population partitioned by the ten groups defined by deciles of each measure of informativeness.**

**
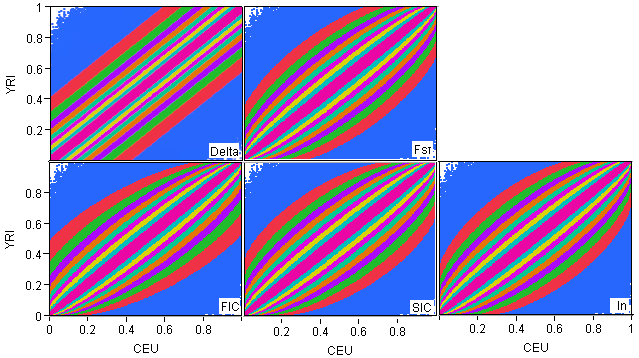
**

The top-left and bottom-right corner represent the most informative SNPs whereas the least informative SNPs reside at the center of the plot.
