# Supplementary material for: Comparison of measures of marker informativeness for ancestry and admixture mapping
Source: BMC Genomics. 2011 Dec 20;12:622. doi: 10.1186/1471-2164-12-622 (PMC3276602; doi:10.1186/1471-2164-12-622)
Supplement: Additional file 6 — Figure S3: Number of AIMs needed to achieve specific accuracies for founder populations. The two founder populations are (a) CEU and YRI and (b) CHB and JPT. [file 1471-2164-12-622-S6.DOCX]

**Additional file 6**

**Figure S3: Number of AIMs needed to achieve specific accuracies for founder populations.**
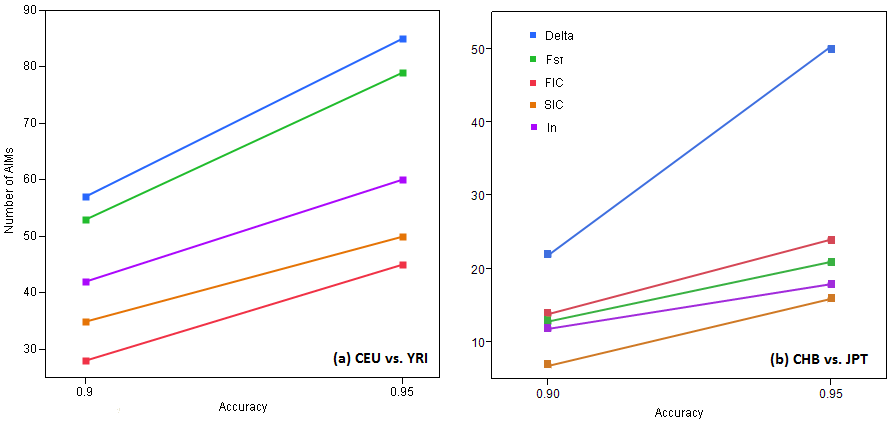


The two founder populations are (a) CEU and YRI and (b) CHB and JPT.
