# Supplementary material for: Comparison of measures of marker informativeness for ancestry and admixture mapping
Source: BMC Genomics. 2011 Dec 20;12:622. doi: 10.1186/1471-2164-12-622 (PMC3276602; doi:10.1186/1471-2164-12-622)
Supplement: Additional file 7 — Figure S4: Inferred population structure for CEU, YRI and ASW population with two clusters and 200 AIMs selected by FIC. A plot of the inferred population structure of CEU, YRI and ASW population. The analysis was done in STRUCTURE and distruct with 2 clusters. [file 1471-2164-12-622-S7.DOCX]

**Additional file 7**

**Figure S4: Inferred population structure for CEU, YRI and ASW population with two clusters and 200 AIMs selected by FIC.**

**
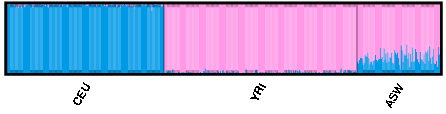
**

The analysis was done in STRUCTURE and *distruct* with 2 clusters.
