# Supplementary material for: Comparison of measures of marker informativeness for ancestry and admixture mapping
Source: BMC Genomics. 2011 Dec 20;12:622. doi: 10.1186/1471-2164-12-622 (PMC3276602; doi:10.1186/1471-2164-12-622)
Supplement: Additional file 8 — Figure S5: Estimate of ancestry contribution vs. number of top AIMs for CEU, YRI and ASW population from HapMap phase III data. Top panel: estimate of CEU contribution for CEU population. Middle panel: estimate of YRI contribution for YRI population. Bottom panel: estimate of YRI contribution for ASW population. [file 1471-2164-12-622-S8.DOCX]

**Additional file 8**

**Figure S5: Estimate of ancestry contribution vs. number of top AIMs for CEU, YRI and ASW population from HapMap phase III data.**


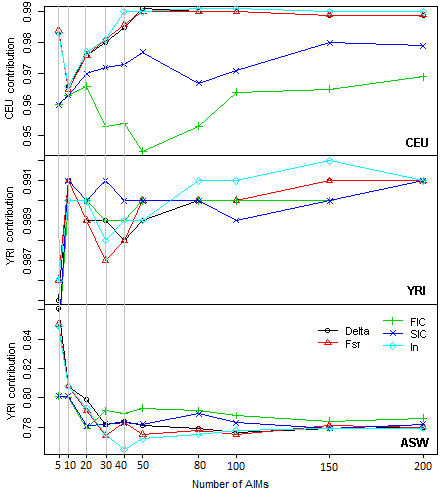


Top panel: estimate of CEU contribution for CEU population. Middle panel: estimate of YRI contribution for YRI population. Bottom panel: estimate of YRI contribution for ASW population.
