# Supplementary material for: Comparison of measures of marker informativeness for ancestry and admixture mapping
Source: BMC Genomics. 2011 Dec 20;12:622. doi: 10.1186/1471-2164-12-622 (PMC3276602; doi:10.1186/1471-2164-12-622)
Supplement: Additional file 9 — Figure S6: Absolute error in the estimation of mean ancestry contribution for the simulated admixed populations. A plot of absolute error in the admixed population simulated from (a) CEU and YRI and (b) CHB and JPT. [file 1471-2164-12-622-S9.DOCX]

**Additional file 9**

**Figure S6: Absolute error in the estimation of mean ancestry contribution for the simulated admixed populations.**


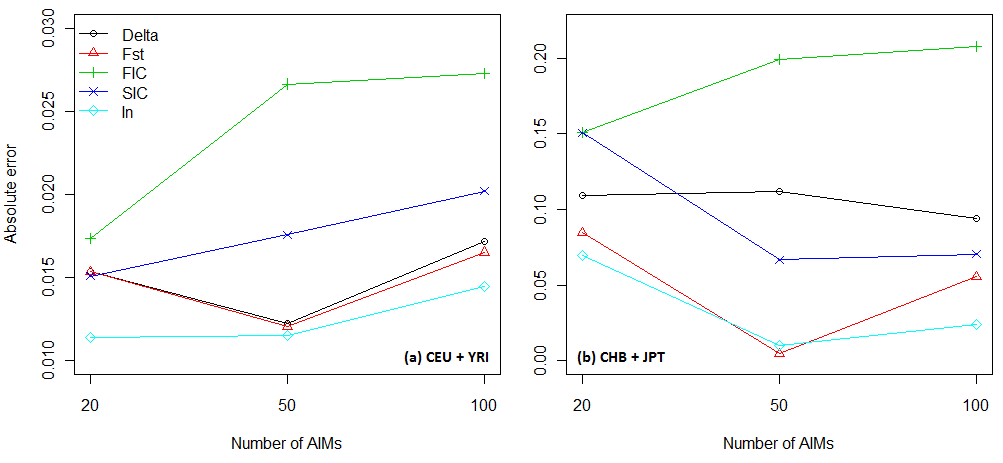


Admixed population simulated from (a) CEU and YRI and (b) CHB and JPT.
