# Supplementary material for: Comparison of measures of marker informativeness for ancestry and admixture mapping
Source: BMC Genomics. 2011 Dec 20;12:622. doi: 10.1186/1471-2164-12-622 (PMC3276602; doi:10.1186/1471-2164-12-622)
Supplement: Additional file 10 — Table S4: Summary statistics of estimation errors of mean ancestry contribution for ASW population. The estimates were based on 100 random subsets of 20 SNPs from panels consisting of top 1%, 2%, 5%, and 10% of the AIMs for CEU and YRI population. The gold-standard or 'true' ancestry contribution was taken as 78%, estimated by a collection of 3299 AIMs for the CEU and YRI population, all of which were selected as top 10% AIMs by at least one of the five measures. [file 1471-2164-12-622-S10.DOCX]

**Additional file 10**

**Table S4: Summary statistics of estimation errors of mean ancestry contribution for ASW population.**

The estimates were based on 100 random subsets of 20 SNPs from panels consisting of top 1%, 2%, 5%, and 10% of the AIMs for CEU and YRI population. The gold-standard or ‘true’ ancestry contribution was taken as 78%, estimated by a collection of 3299 AIMs for the CEU and YRI population, all of which were selected as top 10% AIMs by at least one of the five measures.

| Measure | % | n | Mean | Std Dev | Min | Lower  Quartile | Median | Upper  Quartile | Max |
| --- | --- | --- | --- | --- | --- | --- | --- | --- | --- |
| Delta | 1 | 100 | -0.00536 | 0.015355 | -0.04132 | -0.01309 | -0.00532 | 0.00558 | 0.02777 |
| F_ST_ | 1 | 100 | -0.00493 | 0.013637 | -0.0441 | -0.0134 | -0.00499 | 0.003318 | 0.02423 |
| FIC | 1 | 100 | 0.014745 | 0.012175 | -0.01835 | 0.006816 | 0.01414 | 0.02463 | 0.04299 |
| SIC | 1 | 100 | 0.008812 | 0.011351 | -0.01899 | 0.001877 | 0.01082 | 0.0166 | 0.03293 |
| In | 1 | 100 | -0.00405 | 0.013384 | -0.04049 | -0.0129 | -0.0037 | 0.005482 | 0.0283 |
| AVE | 1 | 100 | 0.002791 | 0.011975 | -0.02338 | -0.00505 | 0.003968 | 0.01073 | 0.02816 |
| MIN | 1 | 100 | 0.008784 | 0.013418 | -0.0247 | -0.00133 | 0.00993 | 0.01864 | 0.04403 |
| Delta | 2 | 100 | -0.00841 | 0.015589 | -0.05237 | -0.01817 | -0.00854 | 0.000255 | 0.0303 |
| F_ST_ | 2 | 100 | -0.00889 | 0.015509 | -0.05519 | -0.02049 | -0.00781 | 0.002066 | 0.0223 |
| FIC | 2 | 100 | 0.018334 | 0.011717 | -0.01581 | 0.01077 | 0.01934 | 0.02669 | 0.04693 |
| SIC | 2 | 100 | 0.008152 | 0.015076 | -0.0325 | -0.00366 | 0.008583 | 0.01705 | 0.05574 |
| In | 2 | 100 | -0.00356 | 0.016035 | -0.03543 | -0.01474 | -0.00299 | 0.004179 | 0.04693 |
| AVE | 2 | 100 | 0.002198 | 0.015283 | -0.03723 | -0.00757 | 0.001276 | 0.01382 | 0.03032 |
| MIN | 2 | 100 | 0.009382 | 0.014966 | -0.02552 | -0.00179 | 0.00923 | 0.0194 | 0.04955 |
| Delta | 5 | 100 | -0.01102 | 0.019738 | -0.07418 | -0.02325 | -0.0117 | 0.001107 | 0.03476 |
| F_ST_ | 5 | 100 | -0.00799 | 0.016114 | -0.03934 | -0.01969 | -0.00869 | 0.001231 | 0.0357 |
| FIC | 5 | 100 | 0.015138 | 0.012497 | -0.01906 | 0.005616 | 0.01564 | 0.02446 | 0.04464 |
| SIC | 5 | 100 | 0.005756 | 0.014719 | -0.03636 | -0.00391 | 0.004197 | 0.0152 | 0.04621 |
| In | 5 | 100 | -0.00566 | 0.016613 | -0.05014 | -0.01656 | -0.00626 | 0.003041 | 0.04425 |
| AVE | 5 | 100 | 0.000522 | 0.016347 | -0.0453 | -0.01119 | 0.00156 | 0.01257 | 0.04539 |
| MIN | 5 | 100 | 0.004451 | 0.015038 | -0.03146 | -0.00466 | 0.004416 | 0.01579 | 0.03521 |
| Delta | 10 | 100 | -0.01356 | 0.018729 | -0.06582 | -0.02545 | -0.01394 | 0.000796 | 0.02623 |
| F_ST_ | 10 | 100 | -0.01144 | 0.020897 | -0.06987 | -0.02426 | -0.01016 | 0.000666 | 0.04343 |
| FIC | 10 | 100 | 0.014584 | 0.013891 | -0.02517 | 0.005801 | 0.01688 | 0.02308 | 0.04613 |
| SIC | 10 | 100 | 0.008109 | 0.016648 | -0.0315 | -0.00191 | 0.007162 | 0.02051 | 0.06628 |
| In | 10 | 100 | -0.00619 | 0.019181 | -0.0368 | -0.01975 | -0.00853 | 0.006001 | 0.07433 |
| AVE | 10 | 100 | -0.00407 | 0.016433 | -0.03706 | -0.01622 | -0.00667 | 0.006632 | 0.04112 |
| MIN | 10 | 100 | 0.002334 | 0.015361 | -0.03382 | -0.00799 | 0.003313 | 0.01356 | 0.03577 |
