# Supplementary material for: Comparison of measures of marker informativeness for ancestry and admixture mapping
Source: BMC Genomics. 2011 Dec 20;12:622. doi: 10.1186/1471-2164-12-622 (PMC3276602; doi:10.1186/1471-2164-12-622)
Supplement: Additional file 11 — Table S5: Summary statistics of estimation errors of mean ancestry contribution for the simulated admixed population from CEU and YRI. The estimates were based on 100 random subsets of 20 SNPs from panels consisting of top 1%, 2%, 5%, and 10% of the AIMs for CEU and YRI population. The true ancestry contribution was 70%. [file 1471-2164-12-622-S11.DOCX]

**Additional file 11**

**Table S5: Summary statistics of estimation errors of mean ancestry contribution for the simulated admixed population from CEU and YRI.**

The estimates were based on 100 random subsets of 20 SNPs from panels consisting of top 1%, 2%, 5%, and 10% of the AIMs for CEU and YRI population. The true ancestry contribution was 70%.

| Measure | % | n | Mean | Std Dev | Min | Lower  Quartile | Median | Upper  Quartile | Max |
| --- | --- | --- | --- | --- | --- | --- | --- | --- | --- |
| Delta | 1 | 100 | -0.02835 | 0.009269 | -0.05016 | -0.03426 | -0.02943 | -0.02154 | -0.00789 |
| F_ST_ | 1 | 100 | -0.02668 | 0.008752 | -0.04714 | -0.03329 | -0.02586 | -0.02183 | -0.00887 |
| FIC | 1 | 100 | -0.04868 | 0.017916 | -0.1132 | -0.06067 | -0.04835 | -0.03549 | -0.01168 |
| SIC | 1 | 100 | -0.03779 | 0.014722 | -0.08773 | -0.04561 | -0.03571 | -0.02798 | -0.0057 |
| In | 1 | 100 | -0.02295 | 0.008989 | -0.04676 | -0.02773 | -0.02219 | -0.01671 | -0.00264 |
| AVE | 1 | 100 | -0.0389 | 0.011696 | -0.06938 | -0.0454 | -0.03828 | -0.03055 | -0.00853 |
| MIN | 1 | 100 | -0.02883 | 0.01081 | -0.05817 | -0.03626 | -0.02852 | -0.02093 | -0.00486 |
| Delta | 2 | 100 | -0.02809 | 0.01184 | -0.06646 | -0.0354 | -0.02866 | -0.01899 | -0.00094 |
| F_ST_ | 2 | 100 | -0.02701 | 0.010914 | -0.05342 | -0.03323 | -0.02565 | -0.01866 | -0.00071 |
| FIC | 2 | 100 | -0.05975 | 0.016011 | -0.109 | -0.07343 | -0.05867 | -0.04927 | -0.0296 |
| SIC | 2 | 100 | -0.04043 | 0.013945 | -0.07801 | -0.05089 | -0.03951 | -0.02988 | -0.01239 |
| In | 2 | 100 | -0.02603 | 0.012282 | -0.0578 | -0.03337 | -0.02661 | -0.01792 | -0.0017 |
| AVE | 2 | 100 | -0.03749 | 0.014386 | -0.07975 | -0.04512 | -0.03674 | -0.02675 | -0.00811 |
| MIN | 2 | 100 | -0.03602 | 0.014324 | -0.07823 | -0.04438 | -0.0345 | -0.02457 | -0.00175 |
| Delta | 5 | 100 | -0.03328 | 0.01285 | -0.0646 | -0.04103 | -0.03325 | -0.02427 | -0.00224 |
| F_ST_ | 5 | 100 | -0.03296 | 0.012605 | -0.07474 | -0.03917 | -0.03213 | -0.02425 | -0.00626 |
| FIC | 5 | 100 | -0.06158 | 0.018149 | -0.1134 | -0.07488 | -0.06029 | -0.05019 | -0.01966 |
| SIC | 5 | 100 | -0.04349 | 0.01625 | -0.08333 | -0.05201 | -0.04235 | -0.03039 | -0.01541 |
| In | 5 | 100 | -0.02684 | 0.012385 | -0.07952 | -0.03437 | -0.02679 | -0.01839 | -0.0035 |
| AVE | 5 | 100 | -0.04124 | 0.017559 | -0.09369 | -0.05157 | -0.04067 | -0.0294 | -0.00349 |
| MIN | 5 | 100 | -0.03814 | 0.012846 | -0.07164 | -0.04702 | -0.03752 | -0.02875 | -0.00868 |
| Delta | 10 | 100 | -0.03351 | 0.015368 | -0.05788 | -0.04744 | -0.03296 | -0.02194 | 0.006092 |
| F_ST_ | 10 | 100 | -0.03167 | 0.014734 | -0.06802 | -0.04151 | -0.03161 | -0.02086 | 0.00383 |
| FIC | 10 | 100 | -0.06885 | 0.02587 | -0.132 | -0.08537 | -0.06609 | -0.05139 | -0.01725 |
| SIC | 10 | 100 | -0.04803 | 0.017239 | -0.1052 | -0.0593 | -0.04681 | -0.03486 | -0.0131 |
| In | 10 | 100 | -0.03568 | 0.015531 | -0.07287 | -0.04698 | -0.03675 | -0.02441 | 0.002302 |
| AVE | 10 | 100 | -0.04357 | 0.016286 | -0.07743 | -0.05468 | -0.04451 | -0.03332 | -0.00395 |
| MIN | 10 | 100 | -0.04073 | 0.015401 | -0.08548 | -0.05189 | -0.03992 | -0.03029 | -0.0013 |
