# Supplementary material for: Comparison of measures of marker informativeness for ancestry and admixture mapping
Source: BMC Genomics. 2011 Dec 20;12:622. doi: 10.1186/1471-2164-12-622 (PMC3276602; doi:10.1186/1471-2164-12-622)
Supplement: Additional file 13 — Table S7: Overlap of SNP markers between measures. Diagonal (bolded): Number of SNPs genotyped in both populations and satisfying the filtering criteriaa. Upper-triangle: Overlap for the SNP markers. Lower-triangle: Overlap for the top 500 ranked SNP markers. [file 1471-2164-12-622-S13.DOCX]

**Additional file 13**

**Table S7: Overlap of SNP markers between measures.**

Diagonal (bolded): Number of SNPs genotyped in both populations and satisfying the filtering criteria^a^. Upper-triangle: Overlap for the SNP markers. Lower-triangle: Overlap for the top 500 ranked SNP markers.

| N (%) | Delta | F_ST_ | FIC | SIC | I_n_ |
| --- | --- | --- | --- | --- | --- |
| Delta | **19807** | 14981 | 8493 | 11134 | 13081 |
| F_ST_ | 479 | **19796** | 10158 | 13910 | 17269 |
| FIC | 220 | 230 | **19765** | 14939 | 11023 |
| SIC | 319 | 329 | 395 | **19760** | 14780 |
| I_n_ | 424 | 445 | 258 | 354 | **19791** |

^a^criteria: 1) The SNP is shared by both YRI and CEU populations, 2) SNPs with missing frequency less than 10% of the samples, and 3) Every SNP is at least 100 kb from its nearest neighbor.
