# Supplementary material for: Comparison of measures of marker informativeness for ancestry and admixture mapping
Source: BMC Genomics. 2011 Dec 20;12:622. doi: 10.1186/1471-2164-12-622 (PMC3276602; doi:10.1186/1471-2164-12-622)
Supplement: Additional file 14 — Figure S7: Scatter plot of allele frequency difference between CEU and YRI population using current cutoff values for each measure. Markers in red exceeded the cutoff for the measure of informativeness. Similar patterns were observed between FST and In. Delta yielded the largest AIMs panel and included a large number of loci not included by any of the remaining four methods. SIC gave the smallest AIMs panel. [file 1471-2164-12-622-S14.DOCX]

**Additional file 14**

**Figure S7: Scatter plot of allele frequency difference between CEU and YRI population using current cutoff values for each measure.**





Markers in red exceeded the cutoff for the measure of informativeness. Similar patterns were observed between F_ST_ and I_n_. Delta yielded the largest AIMs panel and included a large number of loci not included by any of the remaining four methods. SIC gave the smallest AIMs panel.
