# Supplementary material for: Comparison of measures of marker informativeness for ancestry and admixture mapping
Source: BMC Genomics. 2011 Dec 20;12:622. doi: 10.1186/1471-2164-12-622 (PMC3276602; doi:10.1186/1471-2164-12-622)
Supplement: Additional file 15 — Table S8: FIC - Sensitivity analysis of proportion of ancestry contribution on the selection of AIMs. For a pair of proportions of ancestry contribution (m and m'), we examined overlap patterns between the two top n% AIM panels selected using m and m' in the computation of FIC. Overlap patterns were presented by 11: AIMs selected by both panels; 10: AIMs selected by panel one (m) but not panel two (m'); and 01: AIMs selected by panel two (m') but not panel one (m). Frequency and percentage of each overlap pattern were reported for top 1%, 5%, 10%, and 20% AIMs. Proportion of ancestry contribution considered included 0.1, 0.2, 0.3, 0.4, and 0.5. [file 1471-2164-12-622-S15.DOCX]

**Additional file 15**

**Table S8: FIC - Sensitivity analysis of proportion of ancestry contribution on the selection of AIMs.**

For a pair of proportions of ancestry contribution (m and m´), we examined overlap patterns between the two top n% AIM panels selected using m and m´ in the computation of FIC. Overlap patterns were presented by 11: AIMs selected by both panels; 10: AIMs selected by panel one (m) but not panel two (m´); and 01: AIMs selected by panel two (m´) but not panel one (m). Frequency and percentage of each overlap pattern were reported for top 1%, 5%, 10%, and 20% AIMs. Proportion of ancestry contribution considered included 0.1, 0.2, 0.3, 0.4, and 0.5.

|  |  |  | **Top 1% AIMs** | | **Top 5% AIMs** | | **Top 10% AIMs** | | **Top 20% AIMs** | |
| --- | --- | --- | --- | --- | --- | --- | --- | --- | --- | --- |
| **m** | **m´** | **Overlap** | **Freq** | **%** | **Freq** | **%** | **Freq** | **%** | **Freq** | **%** |
| 0.1 | 0.2 | 11 | 142 | 56 | 706 | 56 | 1420 | 56 | 2899 | 58 |
| 0.1 | 0.2 | 10 | 55 | 22 | 281 | 22 | 555 | 22 | 1051 | 21 |
| 0.1 | 0.2 | 01 | 55 | 22 | 282 | 22 | 556 | 22 | 1054 | 21 |
| 0.1 | 0.3 | 11 | 103 | 41 | 517 | 35 | 1076 | 37 | 2156 | 38 |
| 0.1 | 0.3 | 10 | 94 | 37 | 470 | 32 | 899 | 31 | 1794 | 31 |
| 0.1 | 0.3 | 01 | 94 | 37 | 471 | 32 | 901 | 31 | 1798 | 31 |
| 0.1 | 0.4 | 11 | 75 | 30 | 378 | 24 | 792 | 25 | 1616 | 26 |
| 0.1 | 0.4 | 10 | 122 | 48 | 609 | 38 | 1183 | 37 | 2334 | 37 |
| 0.1 | 0.4 | 01 | 123 | 49 | 612 | 38 | 1188 | 38 | 2344 | 37 |
| 0.1 | 0.5 | 11 | 58 | 23 | 279 | 16 | 570 | 17 | 1225 | 18 |
| 0.1 | 0.5 | 10 | 139 | 55 | 708 | 42 | 1405 | 42 | 2725 | 41 |
| 0.1 | 0.5 | 01 | 139 | 55 | 710 | 42 | 1409 | 42 | 2734 | 41 |
| 0.2 | 0.3 | 11 | 152 | 60 | 774 | 64 | 1572 | 66 | 3099 | 64 |
| 0.2 | 0.3 | 10 | 45 | 18 | 214 | 18 | 404 | 17 | 854 | 18 |
| 0.2 | 0.3 | 01 | 45 | 18 | 214 | 18 | 405 | 17 | 855 | 18 |
| 0.2 | 0.4 | 11 | 121 | 48 | 593 | 43 | 1217 | 44 | 2428 | 44 |
| 0.2 | 0.4 | 10 | 76 | 30 | 395 | 29 | 759 | 28 | 1525 | 28 |
| 0.2 | 0.4 | 01 | 77 | 31 | 397 | 29 | 763 | 28 | 1532 | 28 |
| 0.2 | 0.5 | 11 | 93 | 37 | 459 | 30 | 939 | 31 | 1922 | 32 |
| 0.2 | 0.5 | 10 | 104 | 41 | 529 | 35 | 1037 | 34 | 2031 | 34 |
| 0.2 | 0.5 | 01 | 104 | 41 | 530 | 35 | 1040 | 34 | 2037 | 34 |
| 0.3 | 0.4 | 11 | 164 | 65 | 788 | 66 | 1589 | 67 | 3199 | 68 |
| 0.3 | 0.4 | 10 | 33 | 13 | 200 | 17 | 388 | 16 | 755 | 16 |
| 0.3 | 0.4 | 01 | 34 | 13 | 202 | 17 | 391 | 17 | 761 | 16 |
| 0.3 | 0.5 | 11 | 131 | 52 | 636 | 47 | 1286 | 48 | 2607 | 49 |
| 0.3 | 0.5 | 10 | 66 | 26 | 352 | 26 | 691 | 26 | 1347 | 25 |
| 0.3 | 0.5 | 01 | 66 | 26 | 353 | 26 | 693 | 26 | 1352 | 25 |
| 0.4 | 0.5 | 11 | 158 | 63 | 820 | 71 | 1638 | 71 | 3268 | 70 |
| 0.4 | 0.5 | 10 | 40 | 16 | 170 | 15 | 342 | 15 | 692 | 15 |
| 0.4 | 0.5 | 01 | 39 | 15 | 169 | 15 | 341 | 15 | 691 | 15 |
