# Supplementary material for: Comparison of measures of marker informativeness for ancestry and admixture mapping
Source: BMC Genomics. 2011 Dec 20;12:622. doi: 10.1186/1471-2164-12-622 (PMC3276602; doi:10.1186/1471-2164-12-622)
Supplement: Additional file 16 — Table S9: SIC - Sensitivity analysis of proportion of ancestry contribution on the selection of AIMs. For a pair of proportions of ancestry contribution (m and m'), we examined overlap patterns between the two top n% AIM panels selected using m and m' in the computation of SIC. Overlap patterns were presented by 11: AIMs selected by both panels; 10: AIMs selected by panel one (m) but not panel two (m'); and 01: AIMs selected by panel two (m') but not panel one (m). Frequency and percentage of each overlap pattern were reported for top 1%, 5%, 10%, and 20% AIMs. Proportion of ancestry contribution considered included 0.1, 0.2, 0.3, 0.4, and 0.5. [file 1471-2164-12-622-S16.DOCX]

**Additional file 16**

**Table S9: SIC - Sensitivity analysis of proportion of ancestry contribution on the selection of AIMs.**

For a pair of proportions of ancestry contribution (m and m´), we examined overlap patterns between the two top n% AIM panels selected using m and m´ in the computation of SIC. Overlap patterns were presented by 11: AIMs selected by both panels; 10: AIMs selected by panel one (m) but not panel two (m´); and 01: AIMs selected by panel two (m´) but not panel one (m). Frequency and percentage of each overlap pattern were reported for top 1%, 5%, 10%, and 20% AIMs. Proportion of ancestry contribution considered included 0.1, 0.2, 0.3, 0.4, and 0.5.

|  |  |  | **Top 1% AIMs** | | **Top 5% AIMs** | | **Top 10% AIMs** | | **Top 20% AIMs** | |
| --- | --- | --- | --- | --- | --- | --- | --- | --- | --- | --- |
| **m** | **m´** | **Overlap** | **Freq** | **%** | **Freq** | **%** | **Freq** | **%** | **Freq** | **%** |
| 0.1 | 0.2 | 11 | 196 | 100 | 981 | 100 | 1682 | 75 | 3274 | 71 |
| 0.1 | 0.2 | 10 | 0 | 0 | 0 | 0 | 281 | 13 | 653 | 14 |
| 0.1 | 0.2 | 01 | 0 | 0 | 1 | 0 | 282 | 13 | 655 | 14 |
| 0.1 | 0.3 | 11 | 196 | 100 | 979 | 99 | 1556 | 66 | 2793 | 55 |
| 0.1 | 0.3 | 10 | 0 | 0 | 2 | 0 | 407 | 17 | 1134 | 22 |
| 0.1 | 0.3 | 01 | 0 | 0 | 3 | 0 | 409 | 17 | 1138 | 22 |
| 0.1 | 0.4 | 11 | 196 | 100 | 972 | 98 | 1398 | 55 | 2488 | 46 |
| 0.1 | 0.4 | 10 | 0 | 0 | 9 | 1 | 565 | 22 | 1439 | 27 |
| 0.1 | 0.4 | 01 | 0 | 0 | 11 | 1 | 568 | 22 | 1444 | 27 |
| 0.1 | 0.5 | 11 | 196 | 100 | 862 | 78 | 1275 | 48 | 2243 | 40 |
| 0.1 | 0.5 | 10 | 0 | 0 | 119 | 11 | 688 | 26 | 1684 | 30 |
| 0.1 | 0.5 | 01 | 0 | 0 | 121 | 11 | 691 | 26 | 1689 | 30 |
| 0.2 | 0.3 | 11 | 196 | 100 | 980 | 100 | 1838 | 88 | 3440 | 78 |
| 0.2 | 0.3 | 10 | 0 | 0 | 2 | 0 | 126 | 6 | 489 | 11 |
| 0.2 | 0.3 | 01 | 0 | 0 | 2 | 0 | 127 | 6 | 491 | 11 |
| 0.2 | 0.4 | 11 | 196 | 100 | 973 | 98 | 1680 | 75 | 3126 | 66 |
| 0.2 | 0.4 | 10 | 0 | 0 | 9 | 1 | 284 | 13 | 803 | 17 |
| 0.2 | 0.4 | 01 | 0 | 0 | 10 | 1 | 286 | 13 | 806 | 17 |
| 0.2 | 0.5 | 11 | 196 | 100 | 862 | 78 | 1557 | 66 | 2816 | 56 |
| 0.2 | 0.5 | 10 | 0 | 0 | 120 | 11 | 407 | 17 | 1113 | 22 |
| 0.2 | 0.5 | 01 | 0 | 0 | 121 | 11 | 409 | 17 | 1116 | 22 |
| 0.3 | 0.4 | 11 | 196 | 100 | 975 | 98 | 1805 | 85 | 3575 | 83 |
| 0.3 | 0.4 | 10 | 0 | 0 | 7 | 1 | 160 | 8 | 356 | 8 |
| 0.3 | 0.4 | 01 | 0 | 0 | 8 | 1 | 161 | 8 | 357 | 8 |
| 0.3 | 0.5 | 11 | 196 | 100 | 864 | 78 | 1677 | 74 | 3255 | 71 |
| 0.3 | 0.5 | 10 | 0 | 0 | 118 | 11 | 288 | 13 | 676 | 15 |
| 0.3 | 0.5 | 01 | 0 | 0 | 119 | 11 | 289 | 13 | 677 | 15 |
| 0.4 | 0.5 | 11 | 196 | 100 | 871 | 80 | 1835 | 88 | 3603 | 85 |
| 0.4 | 0.5 | 10 | 0 | 0 | 112 | 10 | 131 | 6 | 329 | 8 |
| 0.4 | 0.5 | 01 | 0 | 0 | 112 | 10 | 131 | 6 | 329 | 8 |
